# Transcriptome-wide analysis of alternative RNA splicing events in Epstein-Barr virus-associated gastric carcinomas

**DOI:** 10.1371/journal.pone.0176880

**Published:** 2017-05-11

**Authors:** Victoria E. S. Armero, Marie-Pier Tremblay, Andréa Allaire, Simon Boudreault, Camille Martenon-Brodeur, Cyntia Duval, Mathieu Durand, Elvy Lapointe, Philippe Thibault, Maude Tremblay-Létourneau, Jean-Pierre Perreault, Michelle S. Scott, Martin Bisaillon

**Affiliations:** 1 Département de biochimie, Faculté de médecine et des sciences de la santé, Université de Sherbrooke, Sherbrooke, Quebec, Canada; 2 Plateforme RNomique, Université de Sherbrooke, Sherbrooke, Quebec, Canada; Florida Atlantic University, UNITED STATES

## Abstract

Multiple human diseases including cancer have been associated with a dysregulation in RNA splicing patterns. In the current study, modifications to the global RNA splicing landscape of cellular genes were investigated in the context of Epstein-Barr virus-associated gastric cancer. Global alterations to the RNA splicing landscape of cellular genes was examined in a large-scale screen from 295 primary gastric adenocarcinomas using high-throughput RNA sequencing data. RT-PCR analysis, mass spectrometry, and co-immunoprecipitation studies were also used to experimentally validate and investigate the differential alternative splicing (AS) events that were observed through RNA-seq studies. Our study identifies alterations in the AS patterns of approximately 900 genes such as tumor suppressor genes, transcription factors, splicing factors, and kinases. These findings allowed the identification of unique gene signatures for which AS is misregulated in both Epstein-Barr virus-associated gastric cancer and EBV-negative gastric cancer. Moreover, we show that the expression of Epstein–Barr nuclear antigen 1 (EBNA1) leads to modifications in the AS profile of cellular genes and that the EBNA1 protein interacts with cellular splicing factors. These findings provide insights into the molecular differences between various types of gastric cancer and suggest a role for the EBNA1 protein in the dysregulation of cellular AS.

## Introduction

Gastric carcinoma (GC) is the second leading cause of cancer-related deaths worldwide [1]. The clinical outcome for patients with GC remains poor, with a 5-year survival rate of only about 20% [2]. Moreover, most patients with GC are diagnosed with advanced stage disease, resulting in a dismal prognosis and highlighting the importance of the identification of diagnostic and prognostic markers [3]. Epstein-Barr virus (EBV) infection is associated with 10% of all GC cases reported worldwide [4]. The molecular characterization of EBV-associated gastric carcinomas (EBVaGC) has shown that EBVaGC exhibit specific clinicopathological features, novel genomic and epigenetic aberrations, and a distinct protein expression profile than that of conventional EBV-negative gastric adenocarcinomas [5]. Moreover, EBVaGC is associated with the expression of specific viral proteins (EBNA1, LMP2A, and secreted BARF1) which have been shown to play important roles in the development of GC [6]. The Cancer Genome Atlas (TCGA) project recently reported a broad molecular classification of GC which resulted in the identification of four subtypes: EBV-positive tumors, microsatellite instable tumors, genomically stable tumors, and tumors with chromosomal instability [7].

Alterations in RNA splicing of cellular genes have been observed in many diseases including GC, and the available data suggest that defects in splicing likely play a role in carcinogenesis [8]. Human cells use alternative splicing (AS) to modify the composition of pre-mRNA transcripts through selection of different exons to be included in mature mRNAs, thereby producing a variability at the proteomic level [9]. These various proteins generated from a single gene can frequently support different or even opposite biological effects. This is clearly demonstrated by the aberrant splicing of the RON gene which encodes for a tyrosine kinase receptor. Exclusion of the RON exon 11 results in a mRNA transcript which encodes a constitutively active receptor, ΔRon, harboring constitutive tyrosine kinase activity and promoting an invasive phenotype [10]. Therefore, it is not surprising that AS is tightly regulated and that variations in splicing patterns have been associated with various human diseases such as cancer [11]. AS alterations can provide selective advantages to tumors, such as angiogenesis, proliferation, cell invasion and avoidance of apoptosis [12]. Recent evidences indicate that some of these splicing alterations can be used as prognostic or diagnostic biomarkers, and the identification of molecules capable of correcting and/or inhibiting pathological splicing events is an imperative issue for future therapeutic approaches [13].

Various studies have shown alterations in the AS patterns of a limited number of specific cellular genes in GC. Examples of aberrantly-spliced genes detected in GC include TACC1 (Transforming, Acidic Coiled-Coil Containing Protein 1) [14], S100A4 [15], HTERT [16] (human telomerase reverse transcriptase), CD44 [17], and MET (or hepatocyte growth factor HGF) [18]. In addition, it has been suggested that differentially expressed isoforms could potentially be exploited as biomarkers for gastric cancer [19]. In the current study, high-throughput RNA sequencing data obtained from TCGA were used to investigate the alterations in the global cellular AS landscape of EBVaGC.

## Materials and methods

### Samples and RNA-seq data analysis

Detailed information on the GC samples can be obtained from the original manuscript describing the comprehensive evaluation of 295 primary gastric adenocarcinomas as part of TCGA project [7]. Essentially, each frozen primary tumour specimen had a companion normal tissue specimen [7]. Adjacent non-tumour gastric tissue was also submitted for a subset of cases [7]. Pathology quality control was performed on each tumour and adjacent normal tissue (if available) [7]. Hematoxylin and eosin (H&E) stained sections from each sample were subjected to pathology analysis to confirm that the tumour specimen was histologically consistent with gastric cancer and the adjacent tissue specimen contained no tumour cells [7].

RNA-Seq samples from TCGA were obtained through the CGHub data portal (https://cghub.ucsc.edu/). Since only BAM files were available, a custom script was used to generate valid FASTQ files. The sequence reads were then aligned on the transcriptome reference sequence database UCSCGene Hg19 using Bowtie v2 aligner (default parameters). The associated gene isoforms were quantified in transcript-per-million (TPM) using RSEM for each sample [20,21]. RSEM utilizes an Expectation-Maximization (EM) algorithm as its statistical model which allows reads mapping to multiple transcripts to be included in the quantification. Alternative splicing events were automatically identified and further quantified using the percent-spliced-in (PSI, Ψ) value based on long (L) and short (S) forms of all splicing events present using the equation below:

$$\Psi =\frac{L}{L+S}$$

For each splicing event in one given gene (cassette-exon, mutually exclusive exons, alternative 5’ and 3’ splice site, etc), a PSI value was computed based on the ratio of the long form on total form (short form and long form) present to determine the inclusion of exon, intron retention, differential splice-site choice, etc. For instance, the long form of a cassette-exon would be its inclusion, and short form would be its exclusion from the mature transcript.

### Statistical analysis

The list of alternative splicing event (ASE) was filtered to retain only data present in at least two replicates for both GC and normal stomach tissue. ASEs with a P-value of less than 0.05 were conserved. To ensure higher stringency, the ASEs were filtered with a cutoff Q-value of less than 0.05. From these ASEs, only those with a difference higher than 10% in PSI were considered biologically relevant. Welch's t-test (Student's t-test with unequal sample sizes and unequal variances) was evaluated through the GNU Scientific Library (GSL) (http://www.gnu.org/software/gsl/) integrated to Perl system analysis for both AS and gene expression data. Finally, false discovery rates (fdr) were determined with the Q-value package in R (https://cran.r-project.org/src/contrib/Archive/qvalue/) according to Storey and Tibshirani [22]. For all other analyses, Graph Pad Prism version 6.05 was used to run statistical analysis.

### Gene expression analysis

Initially, the list of selected genes was filtered to retain only data present in at least two replicates for both GC and normal gastric tissue. To allow higher reproducibility, only transcripts with expression levels higher than one transcript-per-million in either dataset were retained. Fold changes (in base 2 logarithm) were then determined between STAD samples and normal gastric tissues. Q-values were finally evaluated, and results under 0.05 were determined significant.

### Gene ontology analysis

Gene ontology analysis was performed with PANTHER using the database for annotation, visualization, and integrated discovery (DAVID) [23].

### Functional ASE prediction

The splicing patterns of selected genes were visualized Using the FAST-DB or EASANA suite. DNA sequences of representative transcripts presenting short and long isoforms were downloaded and translated into proteins using ExPASy translation tool [24] Predicted proteins were then compared using Multalin (truncation and frameshift event) [25], PFAM (loss or appearance of functional domain) [26], and NLS Mapper (loss or gain of nuclear localization signal) [27].

### PCR validation

Gastroesophageal cancer cDNA Arrays form Origene TissueScan plates (Rockville, MD) were assessed for the expression of the various transcripts using the manufacturer's protocol. The plates contained cDNAs from 6 normal and 42 gastroesophageal cancer tissues. All forward and reverse primers were individually resuspended in 20–100 μM stock solution in Tris-EDTA buffer and diluted as a primer pair to 1 μM in RNase DNase-free water. PCR reactions (10 μl) were performed in 96 well plates on a CFX-96 thermocycler (BioRad). For each PCR run, control reactions were performed in the absence of template and carried out for each primer pair. These reactions were consistently negative. The PCR products were finally analyzed using automated chip-based microcapillary electrophoresis on Caliper LC-90 instruments (Caliper LifeSciences). Both amplicon sizing and quantitation were analyzed using the manufacturer’s software.

### Knockdown of splicing factors and effect on alternative splicing

The siRNAs used to knockdown the expression of specific splicing factors and the methodology used to measure the effect on alternative splicing are described in [28]. Proteins and RNA were extracted from mock-transfected (lipofectamine 2000, Invitrogen) and small interfering RNA (siRNA)-transfected cells 96 h posttransfection. Control siRNAs included the siRNA AllStars Negative Control (Qiagen) which has no homology to any known mammalian gene and the KITLG Trilencer-27 Human siRNA (Origene) which contains three unique 27-mer siRNA duplexes targeting the gene encoding the ligand of the tyrosine-kinase receptor.

### Survival analysis for GC patients

The PROGgeneV2 prognostic biomarker identification tool [29] was used to study the implications of splicing factors gene expression on overall survival of GC patients. The preprocessed dataset from TCGA (including RNA-Seq data and clinicopathological features) was used for analysis. The Cox proportional hazard model was used to calculate hazard ratio and p-value of each parameter, and the median gene expression values were used as bifurcation points. A p-value of less than 0.05 was considered significant.

### EBNA1 Expression and Western blot analysis

Stable HEK293T cell lines expressing the EBNA1 protein were generated. HEK293T cells were first transfected with the pMSCV vector (addgene) encoding for the viral EBNA1 protein. Cells with stable EBNA1 protein expression were selected by Puromycine selection (5ug/ml) for 20 days. To maintain the selection, cells were kept in 3ug/ml Puromycine-DMEM complete medium. Cells were grown, pelleted, resuspended and mixed with 50 μl of SDS loading buffer and heated for 5 min at 95°C. Samples were resolved by electrophoresis on 10-% SDS-polyacrylamide gels and transferred onto a polyvinylidene difluoride (PVDF) membrane. The presence of the EBNA1 protein was detected using a mouse anti-FLAG antibody (Sigma). A mouse anti-actin antibody (Sigma) was also used as a control. Following incubation with primary antibodies, PVDF membranes were washed and incubated with the horseradish peroxidase-conjugated secondary antibody (Amersham Biosciences). Proteins were revealed using the enhanced chemiluminescence (ECL) kit from Amersham Biosciences.

### Immunofluorescence

HEK293T cells stably expressing the EBNA1-FLAG-HA protein were seeded in 24 well plates at a density of 5x10^4^ cells/well. The cells were grown for 24 h and washed twice with PBS and fixed with 4% paraformaldehyde and 4% sucrose for 20 min at 22°C. Cells were permeabilized with 0.15% triton X-100 in PBS for 5 min at 22°C and blocked in 10% normal goat serum (Wisent). The EBNA1 protein was stained with primary mouse anti-HA antibody (Abcam) for 4 h at 22°C. Cells were washed and incubated in the dark for 1 h at 22°C with an Alexa Fluor 488-labelled anti-mouse secondary antibody (Abcam). Nuclei were stained with Hoechst (1 μg/ml) for 15 min at 22°C. Cover glasses were affixed on slides with SlowFade mounting medium (Invitrogen S36937). Epifluorescence microscopy was conducted using a Nikon Eclipse TE2000-E visible/epifluorescence inverted microscope using bandpass filters for Hoechst and Alexa Fluor 488.

### Nuclear extracts preparations and mass spectrometry

Hypotonic lysis of HEK293T cells followed by Dounce homogenization, differential centrifugation, and separation of cytoplasm and nuclei was performed as described before [30]. Nuclear extracts were washed three times with PBS and treated with 20 mg/ml RNase A (Invitrogen) and 200 units DNase RQ1 (Promega) for two hours. Immunoprecipitation of the EBNA1 protein was performed using anti-HA beads (Roche) following the manufacturer’s instructions. LC-MS/MS was performed as described previously [31]. Non-specific binders were determined using the CRAPOME database (Contaminant Repository for Affinity Purification and Mass Spectrometry) [32].

### Co-immunoprecipitation

Nuclear extracts were incubated with anti-HA beads (Roche) and the presence of the EBNA1, hnRNP H1, and GAPDH proteins in nuclear extracts was detected using the following specific primary mouse antibodies: anti-FLAG antibody (Sigma), anti-GAPDH (Abcam), and anti-hnRNP H1 (Abcam). Following incubation with primary antibodies, membranes were washed and incubated with the horseradish peroxidase-conjugated secondary antibody (Amersham Biosciences). Proteins were revealed using an enhanced chemiluminescence (ECL) kit (Amersham Biosciences).

## Results

### Alteration of the cellular AS landscape in EBVaGC

**Fig 1:**
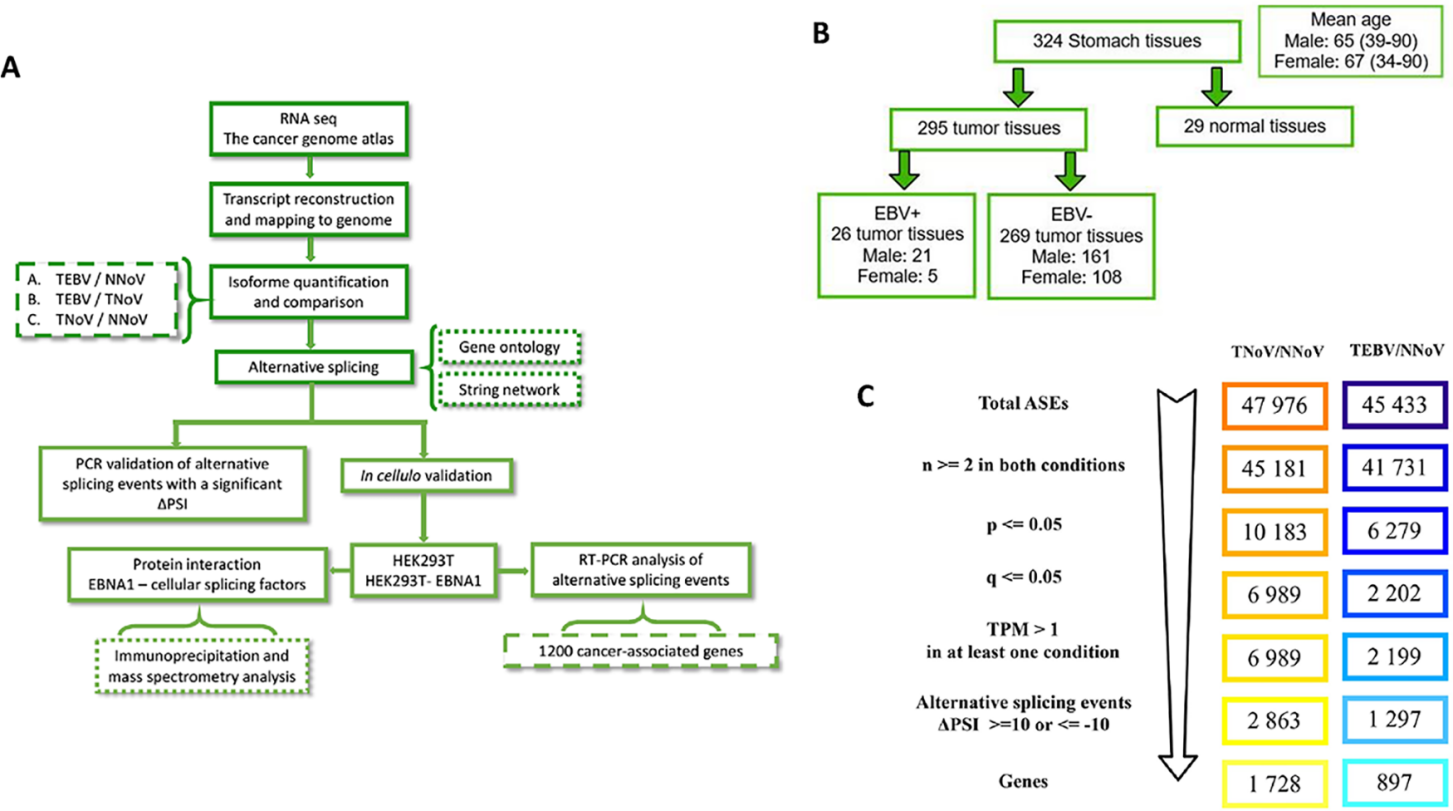
Identification of alternative splicing events in gastric cancer. **(A)** Overview of the strategy used to identify the changes in alternative splicing in GC. **(B)** Classification of the TCGA RNA sequencing data for GC and healthy tissues. **(C)** The splicing events list for both EBV-negative gastric adenocarcinomas (TNoV, Tumors, no virus) and EBV-associated gastric carcinomas (EBVaGC, TEBV, Tumors with EBV) compared the global splicing patterns with normal tissues (NNoV, Normal tissues, no virus) was filtered to keep only significant ASEs.

Modifications to the global RNA splicing landscape of more than 295 primary gastric adenocarcinoma tissues from The Cancer Genome Atlas (TCGA), including data from 26 EBVaGC tissues, were examined using high-throughput RNA sequencing data. Fig 1A and 1B shows an overview of the analyses performed in the current study. RNA-seq data were mapped to the reference genome, followed by transcript assembly and analysis of the various RNA isoforms abundance. We initially focused our study on EBVaGC (EBVaGC, TEBV, Tumors with EBV), and we compared the splicing patterns with normal tissues (NNoV, Normal tissues, no virus). To identify the cellular AS patterns that are modified in EBVaGC, we evaluated the modifications in the relative splice abundances by quantifying all the primary RNA transcripts that produce two or more isoforms. We detected and quantified the various alternative splicing events (ASEs) using the percent-spliced-in (PSI) parameter based on isoform expression (TPM, transcripts per million) for the long and the short isoforms (see Experimental Procedures). The lists of ASEs (47,976 ASEs) were filtered to keep only the transcripts with at least two replicates for both TEBV and NNoV tissues (Fig 1C). Moreover, only significant ASEs (with a P-value of less than 0.05) that differed between tumor and normal gastric tissues were conserved. To provide higher stringency, the ASEs were additionally filtered with a cutoff Q-value (fdr; false-discovery rate) of less than 0.05. From these selected ASEs, only those with a variation higher than 10% in |Delta PSI| were conserved and considered biologically relevant. This strategy allowed the identification of 1297 primary transcripts belonging to 897 genes for which was significantly modified in patients with EBVaGC (Q ≤ 0.05, |Delta PSI| ≥ 10) (Fig 1C). The comprehensive list of the differential ASEs associated with EBVaGC is provided in S1 Table.

**Fig 2:**
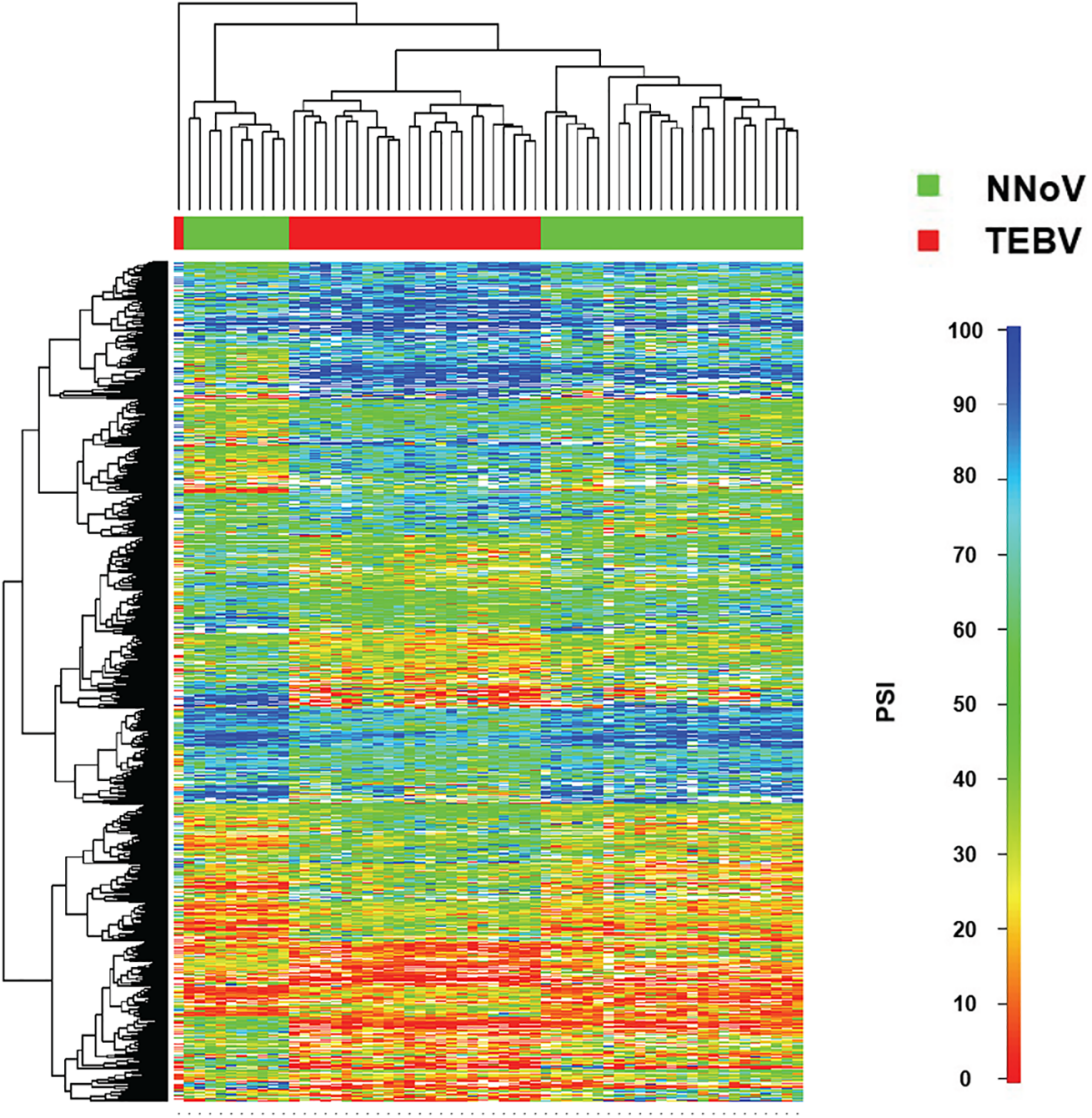
Global profiling of alternative splicing event modifications in EBVaGC. Hierarchical cluster and heatmap representation of alternative splicing events modified in EBVaGC tissues. The row at the top shows the clustering information in the form of a dendogram and the similarity relationships among the alternative splicing events and patients. The column at the left of the heatmap shows the different clusters associated with the PSI values. The heatmap shows PSI values for EBVaGC tissues (TEBV, Tumors with EBV, *in red*) compared with normal tissues (NNoV, Normal tissues, no virus, *in green*).

Our analysis revealed that the AS of numerous cellular transcripts was modified in patients with EBVaGC as compared to normal healthy tissues (Fig 2). Hierarchical clustering clearly distinguished between normal and EBVaGC tissues (Fig 2). Moreover, additional analysis revealed the presence of two distinct clusters for EBVaGC tissues. However, multivariate clustering and statistical relationships among the EBVaGC samples could not detect any correlation between the clusters and clinical parameters (such as age, sex, histological grade, etc.). Further analysis revealed that 198 transcripts had |Delta PSI| values higher than 30% (S1 Fig). To detect cellular pathways for the differential ASEs associated with EBVaGC, we performed a statistical analysis to analyze the overrepresentation of biological processes. The analysis revealed that certain processes such as vesicle-mediated transport, cellular component movement/organization, and metabolic process are statistically overrepresented in the differential ASEs associated with gastric adenocarcinoma (S2 Fig). Through manual curation of functional annotations, we also identified several modifications in the AS patterns of genes potentially involved in oncogenesis (Table 1). Indeed, EBVaGC tissues displayed alterations in the AS patterns of 77 tumor suppressors, 62 transcription factors, and 36 kinases. Some of the dysregulated ASEs that were identified include CD44, which encodes a cell-surface protein implicated in cell-cell interactions, migration, and cell adhesion and which has previously been involved in tumor metastasis and GC [17], and BRCA1 which encodes a nuclear protein involved in genomic stability which also functions as a tumor suppressor and is frequently mutated in breast and ovarian cancers.

**Table 1:** Protein families encoded by transcripts that are differentially spliced in GC.

Protein classification	TNoV / NNoV		TEBV / NNoV	
	Genes	Frequency %	Genes	Frequency %
Extracellular matrix protein (PC00102)	35	2.0%	16	1.8%
Protease (PC00190)	63	3.6%	22	2.5%
Cytoskeletal protein (PC00085)	107	6.2%	70	7.8%
Transporter (PC00227)	115	6.7%	50	5.6%
Transmembrane receptor regulatory (PC00226)	7	0.4%	7	0.8%
Transferase (PC00220)	104	6.0%	63	7.0%
Oxidoreductase (PC00176)	51	2.9%	32	3.6%
Lyase (PC00144)	9	0.5%	8	0.9%
Cell adhesion molecule (PC00069)	55	3.2%	22	2.5%
Ligase (PC00142)	51	2.9%	28	3.1%
Nucleic acid binding (PC00171)	192	11.1%	96	10.7%
Signaling molecule (PC00207)	92	5.3%	45	5.0%
Enzyme modulator (PC00095)	140	8.1%	88	9.8%
Calcium-binding protein (PC00060)	36	2.1%	15	1.7%
Defense/immunity protein (PC00090)	59	3.4%	22	2.5%
Hydrolase (PC00121)	162	9.4%	84	9.4%
Transfer/carrier protein (PC00219)	28	1.6%	14	1.6%
Membrane traffic protein (PC00150)	50	2.9%	30	3.3%
Phosphatase (PC00181)	36	2.1%	19	2.1%
Transcription factor (PC00218)	144	8.3%	62	6.9%
Chaperone (PC00072)	14	0.8%	6	0.7%
Cell junction protein (PC00070)	15	0.9%	14	1.6%
Surfactant (PC00212)	5	0.3%	3	0.3%
Structural protein (PC00211)	5	0.3%	4	0.4%
Kinase (PC00137)	57	3.3%	36	4.0%
Storage protein (PC00210)	3	0.2%	2	0.2%
Receptor (PC00197)	142	8.2%	62	6.9%
Isomerase (PC00135)	9	0.5%	2	0.2%
Splicing factors	6	0.3%	12	1.3%
Tumor suppressors	140	8.1%	77	8.6%

### Characterization of the ASEs that are altered in EBVaGC

We next examined the functional consequences of altered AS on protein function. Among the transcripts for which AS was modified in EBVaGC, many ASEs that we identified affect known protein domains. S2 Table shows the functional consequences of the differentially spliced transcripts on protein function for 48 transcripts that are differentially spliced in gastric adenocarcinoma. Among the differential ASEs, we noted the loss of predicted nuclear localization signals (NLS) for KDM3B (Lysine-specific demethylase 3B), a histone demethylase with potential tumor suppressor activity. Eleven other ASEs were predicted to disrupt known functionally critical protein domains.

### Comparison between EBV-positive and EBV-negative GC

**Fig 3:**
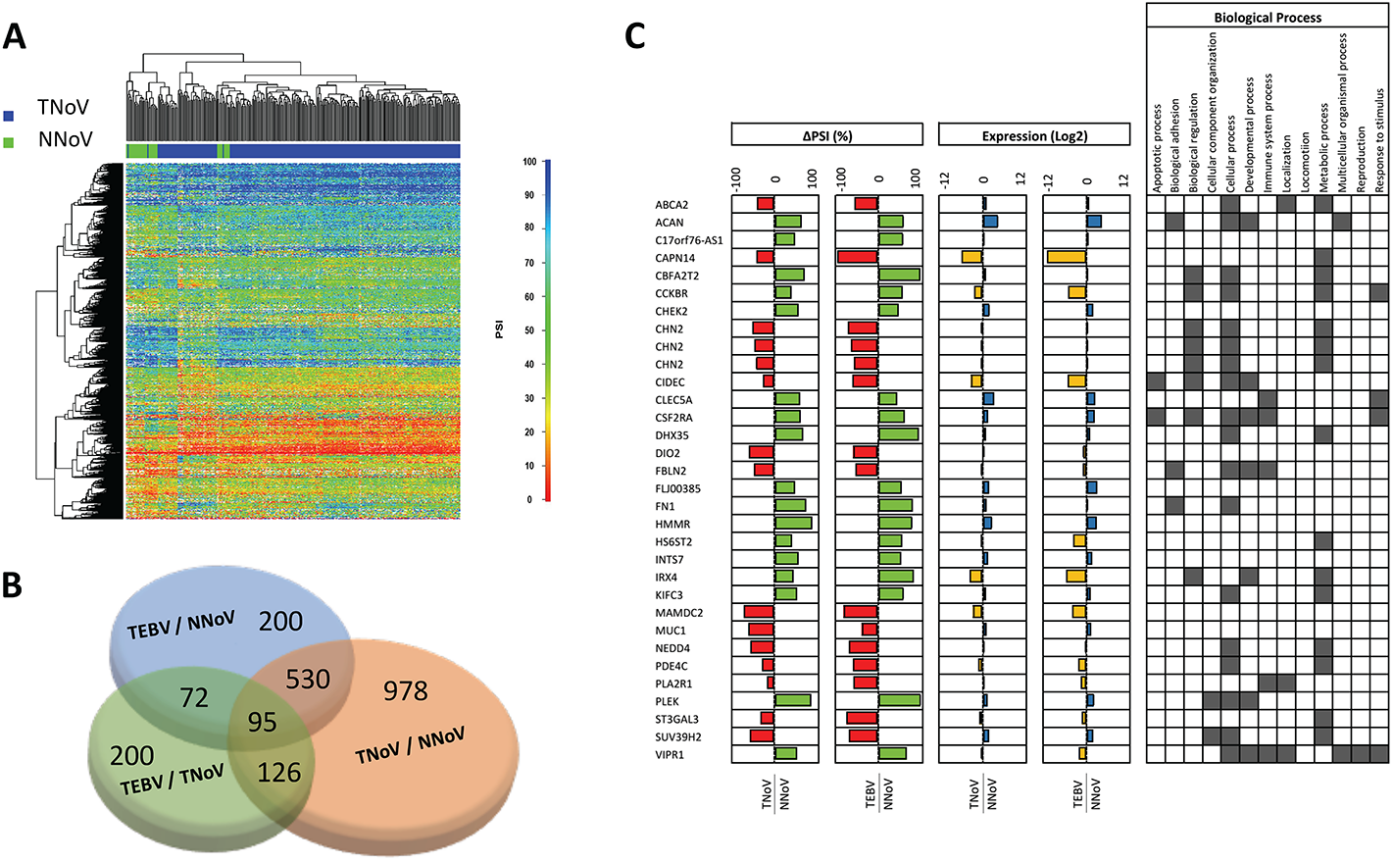
Comparison between EBV-negative and EBV-associated gastric cancer. **(A)** Heatmap representation of isoform ratios (PSI values) for EBV-negative gastric adenocarcinomas tissues. EBV-negative gastric adenocarcinomas tissues (TNoV) are shown in blue, and the comparative healthy tissues are shown in green (NNoV). **(B)** Comparison of the cellular genes with dysregulated ASEs between EBVaGC and EBV-negative GC tissues. **(C)** The list displays common differentially spliced transcripts for both EBV-negative and EBVaGC with the corresponding Delta PSI values, the associated gene expression (in Log_2_), and the related biological processes.

The important number of GC samples analyzed by high-throughput RNA sequencing also allowed us to acquire data for the modifications to the global RNA splicing landscape of EBV-negative gastric adenocarcinomas (TNoV, Tumors, no virus). Our RNA-Seq analysis performed on 269 EBV-negative gastric adenocarcinomas tissues allowed us to identify 2863 primary transcripts belonging to 1728 genes for which the AS pattern was significantly modified in patients with EBV-negative gastric adenocarcinomas in comparison to normal tissues (Fig 1C). A complete list of the differential ASEs associated with EBV-negative gastric adenocarcinomas is provided in S3 Table. Unsupervised clustering on data from EBV-negative gastric adenocarcinomas and integration of these results yielded five distinct clusters associated with modifications in AS (Fig 3A). Since three distinct non-viral subtypes of GC had previously been described following the molecular classification of TCGA (microsatellite instable tumors, genomically stable tumors, and tumors with chromosomal instability), we investigated the possibility that the five clusters associated with EBV-negative gastric adenocarcinomas might be linked to specific molecular subtypes of GC. However, no correlation was found between the non-viral molecular subtypes of GC and the clusters associated with AS, indicating that similar AS changes were occurring in all molecular subtypes. Similarly, we could not detect any correlation between the clusters and clinical parameters (such as age, sex, histological grade, etc.).

The identification of changes in the AS profiles of both EBVaGC and EBV-negative GC tissues raises the possibility that some of the identified ASEs might be a general feature of cancerous tissues. We therefore compared the cellular AS patterns that are altered in EBVaGC (TEBV) with EBV-negative gastric adenocarcinoma (TNoV), and we evaluated the changes in relative splice abundances. Remarkably, 625 genes for which AS was modified in EBVaGC were found to be common with EBV-negative gastric adenocarcinoma tissues (TNoV), suggesting that these ASEs might be a general feature of both types of GC tissues analyzed in this study (Fig 3B, S4 Table). In the case of common misregulated ASEs, the direction of the splicing shift between normal and tumor tissues was frequently the same between the two types of GC (Fig 3C). Using such an approach, we also identified 978 unique genes for which AS is misregulated in EBV-negative gastric adenocarcinoma, while 200 are uniquely misregulated in EBVaGC (Fig 3B, S2 and S3 Tables).

To identify cellular pathways which could be common or different between EBVaGC with EBV-negative gastric adenocarcinoma, we again performed a statistical analysis to analyze the overrepresentation of biological processes. Analysis of the data revealed that certain processes such as vesicle-mediated transport, cellular component movement/organization, and metabolic process are overrepresented for the differential ASEs associated with both EBV-negative GC and EBVaGC (S2 Fig). However, some processes are clearly unique to the differential ASEs associated with EBV-negative GC such as immune system process and intracellular protein transport. The differential ASEs associated with EBV-negative GC also displayed an enrichment for genes involved in heart development although the significance of this finding is not clear.

### Experimental validation on gastric adenocarcinoma tissues

**Fig 4:**
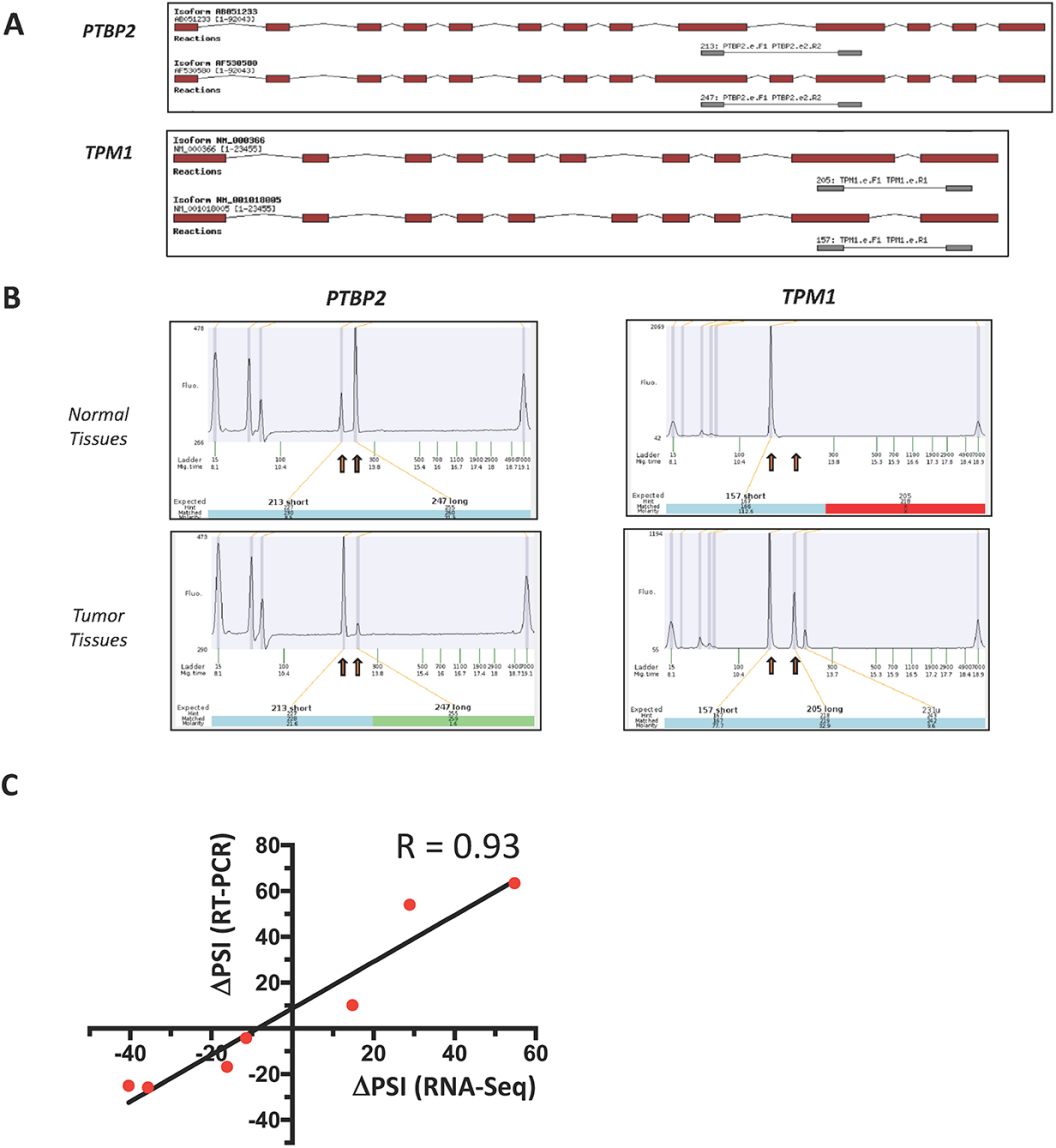
Validation of ASEs dysregulated in gastric cancer. **(A)** Overview of the two isoforms encoded by *PTBP2* and *TPM1* genes. The exons are represented in red and the intervening introns are displayed as thin black lines (not to scale). The primers used to detect the isoforms by RT-PCR are presented in gray and the sizes of the expected amplicons are also presented. **(B)** The cDNAs acquired from gastric tissues were analyzed by PCR using specific primers to detect both isoforms of the transcripts encoded by the *PTBP2* and *TPM1* genes. Capillary electrophoregrams of the PCR reactions are presented. The positions and the amplitude of the detected amplicons are emphasized by red boxes. The positions of the internal markers are also indicated. **(C)** Correlation between PSI values obtained from RNA-Seq and RT-PCR data. The analysis was performed on seven selected ASEs (CAPN14, PLA2G4F, PTBP2, S100A1, SLC52A1, SOGA2, and TPM1). In all cases, the changes in AS levels detected by RT-PCR and the ones revealed through transcriptome sequencing displayed high levels of correlation (R = 0.93).

Analysis of high-throughput RNA sequencing data revealed numerous alterations in the global cellular AS landscape of primary gastric adenocarcinomas. To experimentally confirm the results that were acquired from the RNA-Seq data, we next used PCR analysis from cDNA arrays derived from both healthy and cancerous tissues. Specific primers were designed to allow detection of predicted ASEs by PCR. Two examples of differentially spliced transcripts are presented in Fig 4A illustrating the modifications in isoform usage in transcripts encoded by the *PTBP2* and *TPM1* genes. Our PCR results demonstrated that changes in AS levels that were detected through transcriptome sequencing could also be detected by PCR analysis (Fig 4B). The validation analysis was performed on seven selected ASEs (CAPN14, PLA2G4F, PTBP2, S100A1, SLC52A1, SOGA2, and TPM1) and showed that changes in AS levels detected by RT-PCR were similar to the ones obtained through transcriptome sequencing, and demonstrated high levels of correlation (R = 0.93) (Fig 4C, S3 Fig). It should be noted that Sanger sequencing was also performed on several PCR products to validate the predicted ASEs (data not shown).

### Expression levels and AS of RNA splicing factors

**Fig 5:**
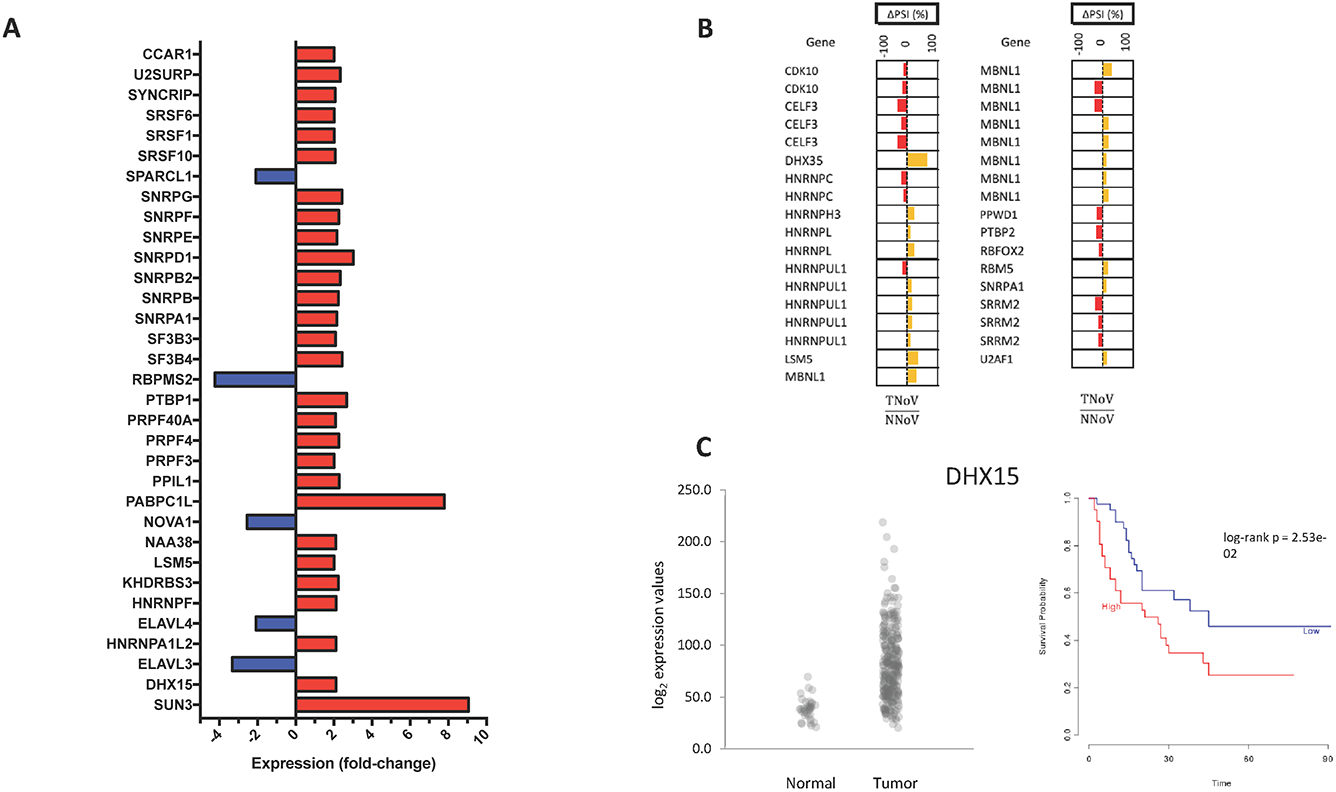
RNA splicing factors in gastric cancer. **(A)** Changes in expression profile of splicing factors in GC tissues (TNoV, Tumors, no virus) compared with the expression in normal tissues. Genes encoding splicing factors for which expression levels were modified by more than 2-fold are presented. *Red* indicates an increase in gene expression and *blue* indicates a decrease in gene expression **(B)** Misregulation of splicing factors alternative splicing in GC (TNoV, Tumors, no virus). The delta PSI values are represented in red (negative delta PSI values) and in yellow (positive delta PSI values). **(C)** Expression levels of DHX15 in patients with or without tumors (left panel). Kaplan-Meier overall survival curve (right panel) for patients expressing high (red) or low (blue) levels of DHX15.

The molecular mechanisms which lead to a modification of the AS landscape in GC are currently unknown. However, aberrant splicing is frequently associated with dysregulated expression of individual splicing factors [33,34]. We therefore monitored both the expression levels and the changes in splicing patterns of RNA encoding for splicing factors and spliceosomal proteins. Although the differential expression of splicing factors was not significantly higher than any other set of genes, the expression of an important number of splicing factors and spliceosomal proteins is indeed affected in GC (Fig 5A, S4A and S5 Figs). RNA-Seq data revealed that the expression of splicing factors was clearly more affected in EBV-positive GC tissues. Indeed, the expression of 33 and 67 proteins involved in splicing was modulated by more than 2-fold in EBV-negative GC and EBVaGC, respectively. We also investigated modifications to the AS patterns of RNA transcripts encoding proteins involved in splicing (splicing factors and proteins of the spliceosome). Our analysis led to the identification of numerous splicing factors that were differentially spliced in GC (Fig 5B, S4B Fig). In the case of EBV-negative gastric adenocarcinoma, the AS patterns of 16 splicing factors were significantly modified. One example is RBFOX2, an RNA binding protein which is thought to be an important regulator of alternative exon splicing. Other examples include CELF3, an important regulator of AS, and MBNL1, which can act either as a repressor or an activator of splicing on specific pre-mRNA targets.

**Fig 6:**
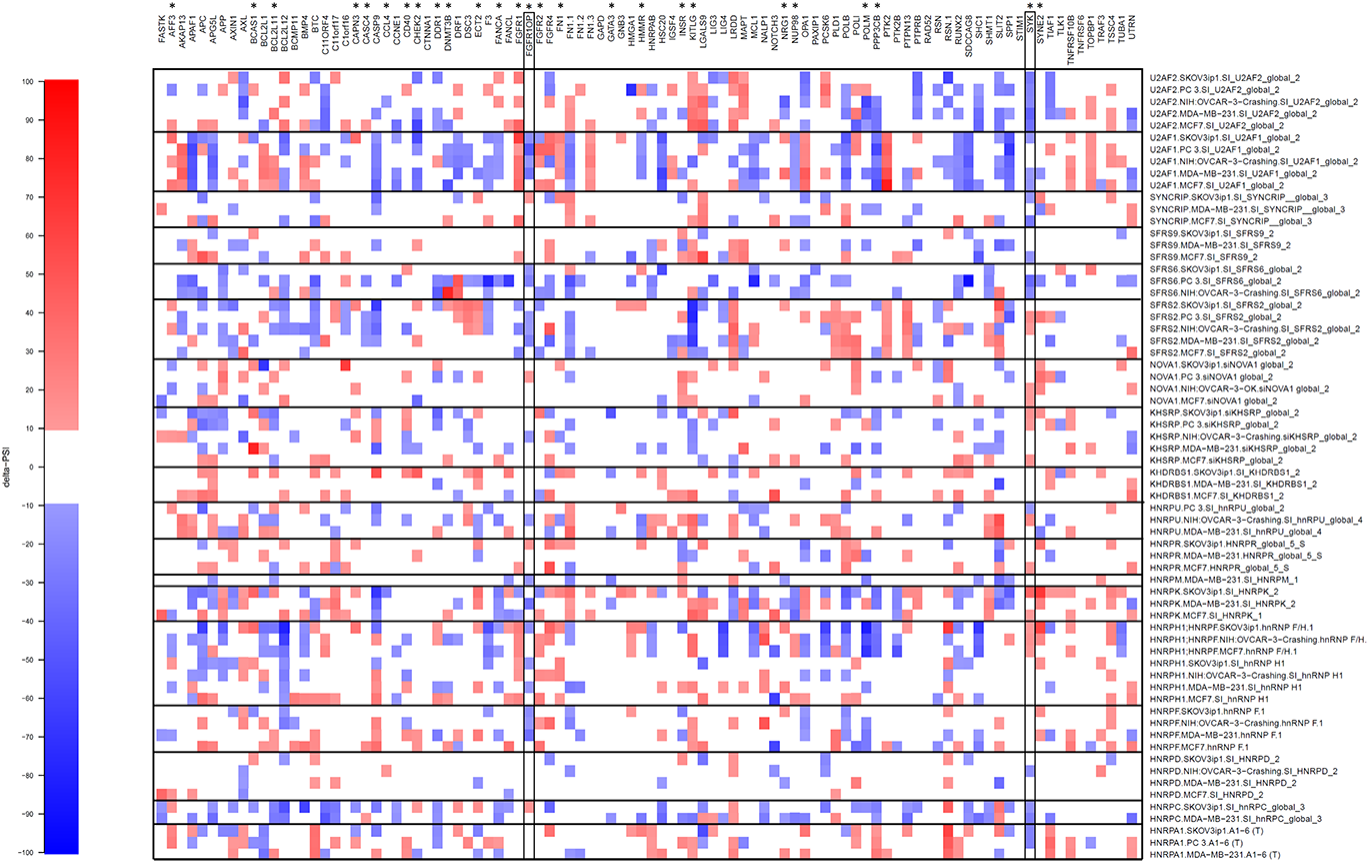
Modifications to AS of 96 transcripts in response to knockdown of specific splicing factors with siRNAs. Using specific siRNAs, eighteen splicing factors (U2AF2, U2AF1, SYNCRIP, SFRS9, SFRS6, SFRS2, NOVA1, KHSRP, KHDRBS1, HNRPU, HNRPR, HNRPM, HNRPK, HNRPH1, HNRPF, HNRPD, HNRPC, and HNRPA1) were individually knocked-down in various cell lines to evaluate their implication in splicing of 96 different transcripts. Asterisks (top) indicate transcripts for which AS was altered in GC. Individual knockdowns and ASEs are presented to indicate which knockdowns produced a shift in AS in various cell lines (PC-3, SKOV3, NIH:OVCAR-3, MDA-MB-231, MCF7). Each individual column represents a different knockdown performed with specific siRNAs. The changes in PSI values are indicated. The map displays the changes in PSI values in a color-coded scale. White areas indicate no shifts.

We further examined the importance of splicing factor levels on AS by using both RNA interference and a reverse transcription-PCR screening platform to analyze the role of splicing factors in different cell lines [28]. We selected various splicing factors, including some which were over- or under-expressed in GC (both EBV-negative GC and EBVaGC). These splicing factors were targeted with specific siRNAs, and we performed a loss-of-function analysis in various cell lines (PC-3, SKOV3, NIH:OVCAR-3, MDA-MB-231, MCF7). Specific siRNAs were therefore designed against various splicing factors (U2AF2, U2AF1, SYNCRIP, SFRS9, SFRS6, SFRS2, NOVA1, KHSRP, HNRPA1, KHDRBS1, HNRPU, HNRPR, HNRPM, HNRPK, HNRPC, HNRPH1, HNRPF, and HNRPD). Depletion efficiencies were determined 96 h posttransfection using Western blotting and/or quantitative RT-PCR assays. Every assay demonstrated that depletion had been efficiently achieved (see Supplemental Data of [28]). Ninety-six transcripts which belong to a subset of apoptotic geneswere selected since the functional consequences of AS on apoptosis have previously been abundantly demonstrated [35]. Twenty-six of these transcripts (AFF3, BCAS1, BCL2L11, CAPN3, CASC4, CCL4, CD40, CHEK2, DDR1, DNMT3B, ECT2, FANCA, FGFR1, FGFR1OP, FGFR2, FN1, GATA3, HMMR, INSR, KITLG, NRG1, NUP98, POLM, PPP3CB, SYK, and SYNE2) were of interest since their AS was altered in GC. The results are depicted in Fig 6 and show that the impact of the individual knockdowns varies considerably. For instance, the knockdown of U2AF1 has a definitive impact on the AS pattern of APAF1, APAPG5L, CASP9, FGFR1, FN1.1, FN1.3, HSC20, MCL1, NUP98, OPA1, POLB, PPP3CB, PTK2, RUNX2, SDCCAG8, SPP1, and TOPBP1 transcripts in all cell lines tested. However, the same knockdown has no impact on the AS profile of numerous other transcripts. Overall, this assay could not specifically correlate the expression of splicing factors which were over- or under-expressed in GC with specific AS modifications observed in GC tissues. However, it demonstrated that the expression of individual splicing factors has an impact on AS profiles, but cannot explain all the changes observed in GC. It should be noted that the use of negative siRNA controls (such as the siRNA AllStars Negative Control and the KITLG Trilencer-27 siRNA) or transfection with lipofectamine alone had no impact on the AS profiles of the 96 transcripts belonging to a subset of apoptotic genes. Not a single modification to the AS profiles of the selected transcripts could be observed (data not shown).

### Expression pattern of DHX15 significantly correlates with patient survival

To explore the potential clinical significance of our findings, we next examined the potential link between the mRNA expression levels of protein factors implicated in splicing and patient survival. We used the recently published PROGgeneV2 prognostic biomarker identification tool [29] to study the implications of splicing factors gene expression on overall survival of GC patients. Kaplan-Meier survival analysis was carried out to determine overall survival for patients as a function of the mRNA expression levels of proteins involved in splicing. Of all the proteins involved in splicing for which the expression level was modulated by more than 2-fold in both EBV-negative GC and EBVaGC, the expression level of DHX15, an ATP-dependent RNA helicase implicated in RNA splicing, showed correlation with patient survival (Fig 5C). A high level of expression of DHX15 was associated with poor patient survival. Using values of median gene expression as bifurcation points, our Cox proportional hazards analyses demonstrated that high expression of this gene was associated with reduced 3-year survival outcomes (p < 0.02). Kaplan-Meier plot indicated significant segregation in survival outcomes for patients with high versus low DHX15 expression. We found no significant correlations between high levels of DHX15 expression and clinicopathological features, including age, gender, and tumor stage. The expression levels of other splicing factors did not appear to be correlated with overall survival.

### Expression of EBNA1 alters cellular AS

**Fig 7:**
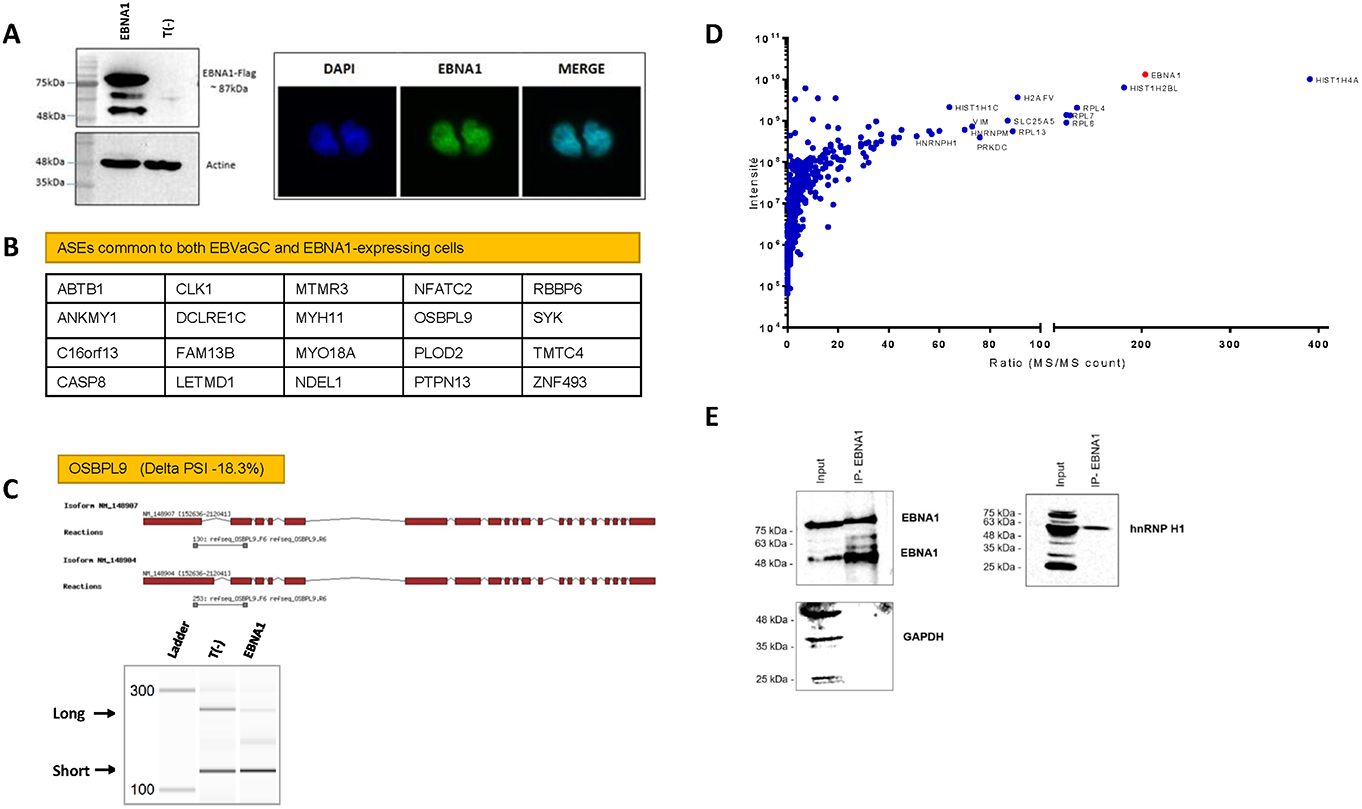
Involvement of EBNA1 in alternative splicing. **(A)** Immunoblotting analysis using anti-HA antibody for the detection of EBNA1-HA-FLAG protein in cell lysates from a stable HEK293T cell line expressing EBNA1. Control HEK293T cells (T(-)) were also used in this assay. **(B)** List of ASEs common to EBVaGC and EBNA1-expressing cells. **(C)** Example of a modified ASE following the expression of EBNA1. Overview of the two isoforms encoded by *OSBPL9* gene. Exons are represented in red and the intervening introns are displayed as thin black lines (not to scale). The primers used to detect the isoforms by RT-PCR assays are presented in gray and the sizes of the expected amplicons are also specified (top panel). RT-PCR reactions were performed on control cells (T(-)) and cells expressing EBNA1 using specific primers to detect both isoforms of the transcripts encoded by the *OSBPL9* gene. Capillary electrophoresis assays were performed and an image of the detected reaction products is presented (lower panel). The positions of the expected amplicons are shown by arrows. **(D)** Mass spectrometry analysis of nuclear proteins interacting with EBNA1. The average ratios (MS/MS counts) of the EBNA1 affinity purification-mass spectrometry experiments were plotted versus the total intensities. **(E)** Validation of the interaction between EBNA1 and splicing factor hnRNP H1. Nuclear extracts were immunoprecipitated with anti-HA. The extracts (input) and immunoprecipitates (IP-EBNA1) were analyzed by immunoblotting and probed with the indicated antibodies.

It is currently not clear whether changes in AS are the cause or the consequence of a malignant phenotype. Moreover, in the case of EBVaGC, it is not clear whether the observed changes in AS result from cellular and/or virus-mediated mechanisms. We therefore investigated the possibility that the presence of a viral protein could contribute to modifications in AS of EBVaGC. We focused on the Epstein-Barr nuclear antigen 1 (EBNA1) protein since EBNA1 is the only viral nuclear protein expressed in EBVaGC, and is also the only viral protein required to maintain viral latency, because of its various roles in the segregation and replication of the EBV episomes [36,37]. There is also increasing evidence that EBNA1 modifies the cellular environment in ways that promote genomic instability, thereby leading to tumorigenesis [38–40]. We relied on a high-throughput reverse transcription-PCR (RT-PCR)-based platform [41] to examine AS in 1200 cancer-associated genes. The stable expression of the EBNA1 protein (harboring a FLAG epitope) was detected by SDS-PAGE in HEK293T cells, a model epithelial cell line extensively used for EBV recombinant molecular genetics [42], followed with anti-FLAG immunoblotting (Fig 7A). HEK293T cells were also chosen because of their high transfection efficiency and their widespread use in RNA splicing studies [43–46]. Nuclear localization of the viral EBNA1 protein was also confirmed by immunofluorescence (Fig 7A). Our results demonstrated that the AS patterns of 112 cellular genes were modified upon EBNA1 expression. Of these, 20 cancer-related genes were also significantly modified in EBVaGC tissues (Fig 7B). Although the presence of these 20 common ASEs between EBNA1-expressing cells and GC tissues is not statistically significative (p-value of 0.29), we observed that the direction of the splicing shift between EBNA1-expressing cells and tumor tissues was the same between the two types of samples (i.e. the sign of PSI variation was identical for all the 20 cancer-related genes identified between the two sample groups). An example of a modified AS pattern which is modified upon EBNA1 expression is provided in Fig 7C. Of the 20 ASEs that are common between cells expressing EBNA1 and EBVaGC, we identified 11 ASEs which do not occur in EBV-negative GC tissues, thereby demonstrating their EBV specificity. The remaining nine other ASEs that were modified (in EBNA1 expressing cells, in EBVaGC tissues and in EBV-negative GC tissues) likely represent ASEs that can be modified in the presence of EBNA1 but can also be modified in GC tissues in the absence of the virus. It should also be noted that in the case of the 112 differential ASEs associated with EBNA1-expressing cells, we only found a significant enrichment for genes involved in response to stimulus (S2 Fig) which probably reflects the fact that cells expressing EBNA1 are clearly not a perfect reflection of EBVaGC tissues.

### EBNA1 interacts with cellular splicing factors

The previous observation that the expression of EBNA1 leads to changes in the AS profile of cellular transcripts led us to examine the capacity of EBNA1 to interact with cellular proteins involved in splicing. We therefore used a quantitative proteomics-based approach using mass spectrometry (LC-MS/MS) to identify cellular proteins that interact with EBNA1. Nuclear extracts of cells stably expressing EBNA1 were prepared and the lysates were treated with DNAse and RNase to remove potential non-specific interactions of EBNA1 with splicing factors (see Materials and Methods for experimental details). Using such an approach, we identified numerous splicing factors which were highly associated with EBNA1 (Fig 7D). These include hnRNP M, hnRNP H1, hnRNP U, hnRNP K, hnRNP C, DHX15, and SFPQ (S4 Table). The physical interaction between EBNA1 and some of these splicing factors was also confirmed by co-immunoprecipitation. An example demonstrating the interaction between EBNA1 and hnRNP H1 is presented in Fig 7E.

## Discussion

The current study provides the first comprehensive portrait of global modifications in the cellular RNA splicing signatures that occur in EBVaGC. Our study showed that the AS patterns of numerous cellular transcripts are modified in EBVaGC. Our data also allowed us to identify unique signatures of genes for which AS is misregulated in EBV-negative and EBVaGC. Analysis of the AS landscape revealed numerous GC–specific markers, which considerably increases the number of potential biomarkers that can presently be recognized using standard expression profiling. The currently available GC markers, such as CEA, CA 19–9 and CA 50, primarily detect advanced GC, for which only palliative treatment is available [3,47] Clearly, future developments in molecular genetics and proteomic analysis are needed to identify other useful GC-specific markers. The current identification of unique signatures for genes in which AS is misregulated in the different types of GC clearly constitutes a step toward that goal.

The expression of numerous cellular splicing factors has been shown to be dysregulated in various human diseases. For example, the expression of the Serine/Arginine-Rich Splicing Factor 1 (SRSF1) is increased in several tumor types [48], and fibroblasts overexpressing SRSF1 can cause tumors when injected into mice [49]. Similarly, an elevated expression of the splicing factor hnRNP H has been demonstrated to drive splicing switches of oncogenic target genes in gliomas [50]. More recently, RBM4, an RNA-binding factor involved in AS, was also shown to control cancer-related splicing events, thereby affecting various cellular processes such as proliferation, apoptosis, and migration [51]. In the present study, we detected many changes in the expression levels and/or splicing patterns of cellular splicing factors in GC tissue samples. We also observed modifications to the AS profiles of many cellular splicing factors including RBFOX2 and MBNL1. How the altered expression/splicing dynamics of these splicing regulators contribute to AS homeostasis during GC remains to be investigated. Interestingly, recent data points to a key role for RBFOX2 and MBNL1 who seem to account for numerous splicing alterations in various cancers including breast, lung, and prostate cancer [52].

Very limited studies have been conducted to find prognosis markers for GC. The most critical prognostic factor influencing overall survival of patients with GC remains the extent of disease as assessed by tumor stage [3]. Indeed, 80% of patients who undergo gastrectomy with stage I disease are alive after 5 years, but only 7% of patients with stage IV disease which has spread to other organs reach the 5-year survival mark. Lymph nodes ratio (ratio of involved to the total resected lymph nodes) has also been shown to have prognostic significance [53]. Moreover, the histological type of the tumor is frequently considered as an essential prognostic factor in GC [3]. In recent years, numerous expression profiles based on the abundance of mRNAs have been put forward as predictors of prognosis in various cancers [54]. For instance, the mRNAs levels of hnRNP K, an important member of the hnRNP family of proteins, was previously shown to be increased in various cancers [55,56]. It has also been reported that high levels of hnRNP K are associated with poorer overall survival of patients with nasopharyngeal carcinoma (NPC) and prostate cancer [55–56]. High levels of expression of another member of the family, namely hnRNP D, is also correlated with reduced survival in oral cancer [57]. In our study, high levels of DHX15 mRNAs were also associated with reduced patient survival in GC. The exact mechanism by which elevated levels of DHX15 mRNAs might result in poor patient survival is currently unknown. However, a previous study on cerium oxide nanoparticles (CNPs) provides a potential link between DHX15 and poor patient survival [58]. CNPs can interfere with cancer development by inducing both cytotoxicity and oxidative stress in cancer cells. The authors of the study demonstrated that treatment of gastric cells with CNPs resulted in an increased expression of DHX15 [58]. This increased expression of DHX15 was shown to activate p38 MAPK signal pathway, leading to the inhibition of proliferation and metastasis in GC both *in vitro* and *in vivo*. It was suggested that CNPs may be a promising approach to suppress malignant activity of GC by increasing the expression of DHX15 [58]. Clinical validation of potential tumor markers such as DHX15 will clearly continue to be a major focus of GC research in the next few years. Indeed, improvements in early diagnosis are still needed since only a minority of patients will be cured of gastric cancer with surgery alone. The identification of valid biomarkers and/or prognosis markers for GC is critical since patients for whom curative resection is not possible generally develop deadly symptomatic metastatic disease from unresected microscopical tumor remnants [2].

Many viruses perturb host cell nuclear functions, presumably to promote an ideal environment for viral replication. These modifications on host cell nuclear functions can be achieved by a variety of mechanisms such as alteration of nuclear architecture [59–62], disruption of nucleocytoplasmic transport pathways [63–66], and induction of nuclear herniations [67]. The exact mechanism by which viral infection leads to alterations of the cellular AS landscape is currently unknown. Two scenarios can be envisaged. First, the cells could respond to viral infection by modifying various pathways which ultimately lead to changes in the AS landscape of cellular RNA. Second, viral factors could potentially disrupt cellular pathways which could lead to a modification of the AS profiles of cellular genes. Recent data indicate that important changes can be observed in the AS patterns of two cellular pre-mRNAs (i.e. PCBP2 and DST) following sequestration of the cellular HuR protein by Sindbis virus [68]. In addition, the Poliovirus protease 2A (2Apro) also induces a nucleo-cytoplasm translocation of numerous RNA binding proteins, including splicing factors, which might result in changes in AS [69]. Moreover, previous studies reported the inhibition of pre-mRNA splicing by the Herpes simplex virus 1 (HSV-1) ICP27 protein [70,71]. In fact, ICP27 has been shown to interact with components of the splicing apparatus, thereby causing a redistribution of splicing factors [72,73]. However, a more recent transcriptomic found no evidence for a generalized inhibition of cellular splicing during HSV-1 infection [74]. The current study demonstrated that the AS patterns of 112 cellular genes (including 20 cancer-related genes which were modified in EBVaGC) were significantly modified upon EBNA1 expression. Interestingly, a previous study demonstrated that the EBV noncoding RNA (EBER1) can interact with splicing factor AUF1/hnRNP D, which leads to variations in cellular AS patterns [75]. These findings suggest that numerous viral factors, in addition to cellular components, are likely involved in the observed alterations in the global cellular AS landscape of EBVaGC. Additional studies will clearly be required to investigate the potential role(s) of additional EBV gene products, such as LMP2A, EBERs, and BARF1, in the modifications of cellular AS during viral infection.

Recent advances in large-scale studies have revealed the extent of modifications that occur in AS in various types of cancer [11]. Although the precise molecular mechanisms which lead to a modification of the AS landscape in GC are currently unknown, our study showed that the expression of the viral EBNA1 protein can lead to alterations in the AS profiles of cellular genes. Our results demonstrated that the AS patterns of 20 cancer-related genes which were modified in EBVaGC were also significantly modified upon EBNA1 expression, thereby suggesting a potential mechanism by which the presence of a virus can modify cellular AS profiles. Numerous direct and/or indirect molecular mechanisms by which the expression of a viral protein such as EBNA1 can lead to a modification of the cellular AS landscape can be envisaged. However, the demonstration that EBNA1 can interact with various splicing factors, as demonstrated by our mass spectrometry studies, clearly constitutes a step towards identifying the mechanisms leading to the extensive AS modifications observed in GC. In addition, the interaction of EBNA1 with splicing factors, many of which have additional roles unrelated to splicing, most likely results in alterations of additional biological processes.

Although changes in splicing patterns are characteristics of cancer development and progression, it is currently not clear whether these modifications are the cause or the result of a malignant phenotype. We are still at an early stage of characterizing the full repertoire of cancer-associated alternatively spliced isoforms in various cancers. In the case of our study on GC tissues, numerous additional functional studies will be required to demonstrate the role, if any, of the identified AS events in carcinogenesis. Moreover, additional layers of complexity are most likely involved. For instance, the AS of tenascin-C is triggered by low pH in normal cells, and this is an effect of nearby tumor growth [76]. Therefore, tumor cells may use this heterotypic signaling with their environment to promote invasion and metastasis. As a result, AS appears intimately bound with cancer cells both as a cause and a consequence [76].

## Supporting information

S1 Table Bioinformatical prediction of functional changes caused by some identified ASEs. Negative delta-PSI indicates that the short isoform is favored and positive delta-PSI indicates that the long isoform is favored.
(PDF)

S2 Table List of genes for which AS is uniquely dysregulated in EBVaGC. (PDF)

S3 Table List of genes for which AS is uniquely dysregulated in EBV-negative GC (Tumor, no virus). (PDF)

S4 Table List of splicing factors that co-purify with EBNA1. (PDF)

S1 Fig Characterization of splicing mysregulation in gastric cancer. Distribution of Delta PSI values in GC.
(PDF)

S2 Fig Statistical analysis for the overrepresentation of biological processes. Overrepresentation of biological processes was analyzed for the differential ASEs associated with EBV-negative GC, EBVaGC, and cells expressing the EBNA1 protein. The first column highlights the name of the biological process, the second column displays the total corresponding number of genes in the database (PANTHER), the third column highlights the number of genes from the searched sample, the fourth column displays the expected number of genes in a normal cell, and the fifth column displays the fold-enrichment. The p-value associated with each overrepresentation is also displayed (last column).
(PDF)

S3 Fig Validation of ASEs dysregulated in gastric cancer. The cDNAs acquired from gastric tissues were analyzed by PCR using specific primers to detect both isoforms of the transcripts encoded by the *CARN14*, *PLA2G4F*, *S100A1*, *SLC52A1*, and *SOGA2* genes. Capillary electrophoregrams of the PCR reactions are presented. The positions and the amplitude of the detected amplicons are indicated. The positions of the internal markers are also shown.
(PDF)

S4 Fig RNA splicing factors in EBVaGC. (A) Iris Graph representing the expression profile of splicing factors for EBVaGC. Differences in gene expression levels are shown on a logarithmic color scale (Log2), from red (negative changes in expression) to blue (increase in gene expression). The expression of proteins involved in splicing modulated by more than 2-fold is indicated by an asterisk. (B) Misregulation of splicing factors alternative splicing in GC (TEBV, Tumors with EBV). The delta PSI values are represented in red (negative delta PSI values) and in blue (positive delta PSI values).)
(PDF)

S5 Fig Variations in the expression of RNA splicing factors in gastric cancer. Differences in gene expression levels of proteins involved in splicing are shown. The figure only shows the splicing factors for which the expression level varied by more than 2-fold. A logarithmic color scale (Log2) is used. Red indicates negative changes in expression and blue indicates increase in gene expression.
(PDF)

## Abbreviations

AS: Alternative splicing
ASE: Alternative splicing event
EBV: Epstein-Barr virus
EBVaGC: EBV-associated gastric carcinomas
EBNA1: Epstein-Barr nuclear antigen 1
GC: Gastric carcinoma
NNoV: Normal tissues, no virus
PSI: Percent-spliced-in
TCGA: The Cancer Genome Atlas
TNoV: Virus-free gastric adenocarcinoma tissues
TEBV: EBV-positive gastric adenocarcinoma tissues
TNoV: EBV-negative gastric adenocarcinoma tissues
TPM: Transcript-per-million
