# Supplementary material for: Transcriptome-wide analysis of alternative RNA splicing events in Epstein-Barr virus-associated gastric carcinomas
Source: PLoS One. 2017 May 11;12(5):e0176880. doi: 10.1371/journal.pone.0176880 (PMC5426614; doi:10.1371/journal.pone.0176880)
Supplement: S1 Table — Negative delta-PSI indicates that the short isoform is favored and positive delta-PSI indicates that the long isoform is favored. (PDF) [file pone.0176880.s001.pdf]

S1 Table - Bioinformatical prediction of functional changes caused by some identified ASEs. Negative delta-PSI indicates that the short isoform is favored and positive delta-PSI indicates that the long isoform is favored)

Gene	ΔPSI (%)		ASE type	Protein size variation	Predicted functional consequence
	TEBV/NNoV	TNoV/NNoV			
**ANKRD30BL**		-47.8	exon-cassette	-	Unknown/ Unpredictable change
**APC2**	-40.2	-18.4	alternate-3p	1 aa	Unknown/ Unpredictable change
**AQP12B**	-30.1		exon-cassette	-	Unknown/ Unpredictable change
**C16orf59**	43.5	45.0	alternate-3p	43 aa	Unknown/ Unpredictable change
**CA13**	-10.7		exon-cassette	-	Unknown/ Unpredictable change
**CAPN14**	-95.7	-40.4	exon-cassette	178 aa	Lost of cystéine peptidase domain
**CD47**	29.3	19.7	multiple-exon-cassette	15 aa	Unknown/ Unpredictable change
**CXXC4**		10.2	mutually-exclusive-exon	-	Unknown/ Unpredictable change
**DAB2IP**	-10.6		alternate-3p	567 aa	Lost of Ras GTPase-activating protein domain and partial loss of Rho GTPase-activating domain
**FAM86JP**	-29.6	-21.1	alternate-5p	-	Unknown/ Unpredictable change
**FBLN2**	-52.0	-47.1	exon-cassette	47 aa	Calcium-binding EGF domain loss in short isoform
**HEPACAM**		-19.9	alternate-3p	282 aa	Loss of Immunoglobulin domain and transmembrane domain in short isoform
**HOXB6**		34.2	alternate-3p	0 aa	Same protein in both isoforms
**INMT**		34.3	alternate-3p	1 aa	Unknown/ Unpredictable change
**KCNMB3**		17.9	exon-cassette	-	Unknown/ Unpredictable change
**KDM3B**	-11.0	-10.0	exon-cassette	624 aa	Loss of NLS signal in short isoform
**KIFC1**		17.3	alternate-3p	8 aa	Unknown/ Unpredictable change
**KIR2DL4**		-37.3	exon-cassette	47 aa	Loss of two intermembrane domains in long isoform

S1 Table (cont'd)- Bioinformatical prediction of functional changes caused by some identified ASEs. Negative delta-PSI indicates that the short isoform is favored and positive delta-PSI indicates that the long isoform is favored)

Gene	ΔPSI (%)		ASE type	Protein size variation	Predicted functional consequence
	TEBV/NNoV	TNoV/NNoV			
**L3MBTL4**		-11.1	exon-cassette	10 aa	Unknown/ Unpredictable change
**LY6G5C**	32.8		mutually-exclusive-exon	-	Unknown/ Unpredictable change
**MAMDC2**	-79.3	-70.3	alternate-5p	-	Unknown/ Unpredictable change
**MARVELD3**	31.5	25.6	alternate-5p	262 aa	Unknown/ Unpredictable change
**mir-29b-2**	36.6		exon-cassette	-	Unknown/ Unpredictable change
**MTBP**	-34.1	-28.5	alternate-3p	-	Unknown/ Unpredictable change
**NPM1**		15.5	exon-cassette	29 aa	Unknown/ Unpredictable change
**NPY4R**	35.9	24.6	exon-cassette	-	Unknown/ Unpredictable change
**PAPSS2**		-32.7	exon-cassette	5 aa	Unknown/ Unpredictable change
**PCDH17**		48.2	alternate-3p	1 aa	Unknown/ Unpredictable change
**PFN2**	-18.2		alternate-3p	-	Unknown/ Unpredictable change
**PLA2R1**	-56.7		alternate-5p	2 aa	Unknown/ Unpredictable change
**PLEK**	99.5	84.6	alternate-3p	60 aa	Lost of a PH domain in short isoform
**PMEPA1**		80.3	alternate-5p	15 aa	Lost of intermembrane domains
**PNLIPRP1**		40.1	alternate-3p	-	Unknown/ Unpredictable change
**PPP4C**		13.8	alternate-5p	-	Unknown/ Unpredictable change
**RANBP9**		-12.1	exon-cassette	341 aa	Lost of Concanavalin A-like lectin/glucanase domain and SPRY domain
**S100A1**	34.5	14.8	alternate-3p	-	Unknown/ Unpredictable change

S1 Table (cont'd)- Bioinformatical prediction of functional changes caused by some identified ASEs. Negative delta-PSI indicates that the short isoform is favored and positive delta-PSI indicates that the long isoform is favored)

Gene	ΔPSI (%)		ASE type	Protein size variation	Predicted functional consequence
	TEBV/NNoV	TNoV/NNoV			
**SASS6**	46.0	46.6	exon-cassette	167 aa	Lost of Centriolar protein SAS N-terminal domain in short isoform
**SERPINB3**		-19.1	exon-cassette	52 aa	Partial lack of Serpin (serine protease inhibitor) domain in short isoform
**SHH**	-37.1	-32.0	exon-cassette	-	Unknown/ Unpredictable change
**SLC17A8**		69.3	exon-cassette	50 aa	A Major Facilitator Superfamily domain complete in long isoform but absent in the short isoform
**SYP**	-44.4		exon-cassette	118 aa	Incomplete Membrane-associating domain in short isoform and loss of some intermembrane domains
**TOPORS**		-13.4	exon-cassette	66 aa	Unknown/ Unpredictable change
**UNC5B**	-11.1	-10.4	exon-cassette	11 aa	Unknown/ Unpredictable change
**XAGE1B**		-54.5	alternate-5p	12 aa	Frame shift after the 32aa of protein sequence
**XAGE1C**		-54.5	alternate-5p	12 aa	Frame shift after the 32aa of protein sequence
**XAGE1D**		-54.5	alternate-5p	12 aa	Frame shift after the 32aa of protein sequence
**ZNF181**		76.1	alternate-3p+intron-retention	64 aa	Loss of KRAB box domain in long isoform
**ZNF781**		39.7	exon-cassette	5 aa	Unknown/ Unpredictable change
