# Supplementary material for: Transcriptome-wide analysis of alternative RNA splicing events in Epstein-Barr virus-associated gastric carcinomas
Source: PLoS One. 2017 May 11;12(5):e0176880. doi: 10.1371/journal.pone.0176880 (PMC5426614; doi:10.1371/journal.pone.0176880)
Supplement: S2 Table — (PDF) [file pone.0176880.s002.pdf]

S2 Table – List of genes for which AS is uniquely dysregulated in EBVaGC

TEBV/NNoV				
ABTB1	DAB2IP	IKBIP	NIPBL	SIGLEC8
ACBD4	DBNDD2	IL17RE	NOTCH4	SLC43A2
ALKBH2	DCLRE1C	IL18BP	NPHP4	SMC6
ANKRD13A	DLEU2	IL4R	NPRL3	SNED1
ANKRD16	DLGAP4	IP6K2	NUBP2	SNRNP70
AP2B1	DVL1	ISOC2	OSBP2	SNX20
AP3M1	DYNLRB2	KCNMA1	PAFAH1B3	SNX24
ARHGAP25	EDEM2	KDM3B	PAN3	SOCS2-AS1
ARMCX5	EHBP1	KIAA1524	PANK2	SPG11
ATP5C1	EIF2AK4	LARP1B	PBX3	SPTAN1
ATP5J	EIF4A2	LETMD1	PCNX	SSBP4
ATP9B	ENPP2	LGI1	PDCD10	STAG2
ATXN2L	ENTHD2	LIMK2	PDE4D	STIL
BAALC	ENTPD1	LINC00116	PDHX	SUPT5H
BHLHE40-AS1	ERI2	LINC00875	PDP1	SYK
BRE	ERN2	LOC100506190	PEX19	TARS2
BTBD7	ESYT3	LRP5L	PGF	TBC1D9B
BUB3	FAM114A2	LRPPRC	PHRF1	TCFL5
BZRAP1	FAM122C	LRR1	POLDIP3	TDRD7
C11orf54	FAM13B	LSR	PPHLN1	TK2
C1QTNF9B-AS1	FAM213B	LTA4H	PPP2R3A	TMTC4
C1RL-AS1	FARP2	MAGI1	PRKCSH	TNPO3
CA13	FBXL19	MAGIX	PTPRE	TOM1L1
CACNA1A	FBXO24	MANEAL	PYHIN1	TOP1
CAMK2D	FBXO7	MAPKAP1	QKI	TROAP
CARD9	FGFR1OP	MCAT	R3HCC1L	TSTD1
CCDC57	FLJ39739	ME1	RALGPS1	TTLL3
CD34	FMNL2	METAP1	RALGPS2	UQCRH
CD86	FOXJ3	MGAT4A	RASGRP3	USP33
CD97	FRS2	MPP5	RBBP6	VTI1B
CDC25A	GDPD5	MSR1	REEP2	WIZ
CDC42BPA	GOPC	MX1	RNH1	WNK2
CDH23	GPR161	MXD1	RPL15	YDJC
CDS2	GRIN1	MXRA7	RSPH3	ZFAS1
CEP68	GTF2I	MYOF	RTN2	ZFYVE16
CHURC1-FNTB	GTF3C1	NCBP2	RWDD3	ZNF195
CIITA	HDDC2	NDEL1	SCP2	ZNF326
COPS7A	HERC2P9	NDUFV3	SEMA3B	ZNF534
CRYBB2P1	HPS4	NEURL4	SETD2	ZNF559-ZNF177
CTSL1	IFNLR1	NF1	SGSM2	ZNF569
