# Supplementary material for: Transcriptome-wide analysis of alternative RNA splicing events in Epstein-Barr virus-associated gastric carcinomas
Source: PLoS One. 2017 May 11;12(5):e0176880. doi: 10.1371/journal.pone.0176880 (PMC5426614; doi:10.1371/journal.pone.0176880)
Supplement: S3 Table — (PDF) [file pone.0176880.s003.pdf]

## S3 Table – List of genes for which AS is uniquely dysregulated in EBV-negative GC (Tumor, no virus)

| TNoV/NNoV | | | | | | | | | |
|---|---|---|---|---|---|---|---|---|---|
| ABCB9 | ANK2 | BBX | CAND2 | CDRT1 | CPM | DSCR3 | FAM47E | GK | IFT88 |
| ABCC4 | ANK3 | BCAS1 | CAP2 | CDV3 | CREB3L4 | DTNA | FAM5C | GLIS3 | IGSF1 |
| ABCC5 | ANKLE1 | BCAT2 | CAPN9 | CEACAM20 | CRISP2 | DUOXA1 | FAM60A | GMPR2 | IKBKB |
| ABCG5 | ANKRD12 | BCL2L13 | CAPNS1 | CEACAM21 | CROCC | DYNC1I2 | FAM66C | GNAI2 | IKZF3 |
| ABHD16A | ANKRD30BL | BDP1 | CARD8 | CELF4 | CSMD2 | E2F6 | FAM86B2 | GNE | IL12RB2 |
| ABHD6 | ANKRD9 | BHLHB9 | CARKD | CEP57 | CSNK1G3 | ECE2 | FANCD2 | GPM6A | IL6 |
| ABL2 | ANXA7 | BIRC5 | CARNS1 | CEP95 | CTF1 | EDN2 | FBXL13 | GPR110 | IMPDH1 |
| ABLIM2 | ANXA8 | BLCAP | CASC4 | CERS3 | CTNNA3 | EFCAB1 | FBXO3 | GPR115 | INCENP |
| ACAD8 | AP1G1 | BMF | CASP5 | CES5A | CUL2 | EIF3B | FCAR | GPR133 | INPP5K |
| ACCS | AP4B1 | BNC2 | CAST | CFTR | CXADR | EIF4G2 | FCGR2B | GPR17 | INTS7 |
| ACMSD | APBB1 | BNIPL | CATSPERG | CHD1L | CXXC4 | EIF4G3 | FCRL1 | GPRASP1 | IRAK4 |
| ACP2 | APLP2 | BRD8 | CBLB | CHD5 | CYB561A3 | EIF4H | FCRL2 | GPS1 | IRF5 |
| ACSF2 | APOBEC1 | BRD9 | CBWD3 | CHEK1 | CYFIP2 | ELMOD3 | FCRL6 | GRHL1 | IRX6 |
| ACSL6 | APOL1 | BSDC1 | CCDC117 | CHEK2 | CYP11A1 | ELOVL7 | FDXR | GRIA2 | IST1 |
| ADAM12 | AQP7 | BTN3A1 | CCDC136 | CHRDL1 | CYP1A1 | EMILIN1 | FER | GRIK5 | ITGA7 |
| ADAM33 | AQPEP | BTRC | CCDC151 | CIDEA | CYP2D6 | ENOSF1 | FEZ2 | GRM4 | ITGB3BP |
| ADAMTS10 | ARAF | C11orf30 | CCDC169 | CIT | CYP2E1 | ENPP3 | FGFR3 | GRP | ITLN2 |
| ADAMTS12 | ARAP3 | C12orf28 | CCDC17 | CKMT1A | CYP2R1 | ENSA | FGGY | GSDMB | ITPK1 |
| ADAMTS14 | ARFGAP1 | C14orf105 | CCDC181 | CKMT2 | DAB1 | EP400 | FHOD3 | GSN | KALRN |
| ADAMTS7 | ARFGAP2 | C14orf64 | CCDC43 | CLCA1 | DAND5 | EPB41L3 | FKBP7 | GZF1 | KAT6B |
| ADCY10P1 | ARFGAP3 | C17orf78 | CCDC66 | CLCN5 | DAP3 | EPB41L5 | FOSB | H2AFV | KAT7 |
| ADH1B | ARHGAP17 | C1D | CCDC7 | CLDND1 | DBNL | ERG | FOXN3 | HAL | KBTBD12 |
| ADRA1A | ARHGAP5 | C1orf168 | CCL28 | CLEC18B | DCAF17 | ERGIC3 | FOXRED1 | HARS2 | KCNAB2 |
| ADSSL1 | ARHGEF25 | C2CD5 | CCM2 | CLEC7A | DCBLD1 | ETFDH | FPGT | HAS1 | KCNE1 |
| AFF1 | ARHGEF4 | C3orf18 | CCNB3 | CLN3 | DCLK2 | EXOC1 | FRY | HDAC9 | KCNJ15 |
| AFF3 | ARID1A | C4B_2 | CCNC | CLUHP3 | DCTN1 | EXOG | FSCN2 | HECTD2 | KCNT2 |
| AFG3L1P | ARMC10 | C4orf26 | CCNL2 | CNKSR2 | DCUN1D2 | EZH1 | FSIP2 | HEPACAM | KDM2A |
| AGAP2 | ARNTL | C5orf34 | CCNY | CNOT10 | DDX19A | EZH2 | FTSJD1 | HEPH | KDM8 |
| AK5 | ASMTL | C7orf43 | CCR3 | CNTLN | DDX3X | FAF1 | GABRB2 | HERC3 | KHNYN |
| AKNAD1 | ASPDH | C9orf135 | CD1E | CNTN2 | DDX58 | FAM120A | GABRP | HFE | KIAA0226 |
| AKR1C2 | ATL2 | C9orf84 | CD27-AS1 | CNTN5 | DENND1A | FAM122B | GALNT11 | HKR1 | KIAA1033 |
| ALCAM | ATP4A | CA12 | CD300A | COCH | DEPDC5 | FAM124B | GAP43 | HLA-DQB2 | KIAA1217 |
| ALDH9A1 | ATP5G2 | CA6 | CD300LG | COG3 | DGKB | FAM126A | GATA3 | HNRNPC | KIAA1462 |
| ALDOA | ATP6V0E2 | CABYR | CD46 | COL21A1 | DIP2B | FAM13A | GDPD2 | HNRNPL | KIAA1614 |
| ALOX15B | ATP8A1 | CACNB2 | CD80 | COL4A6 | DKFZp667P0924 | FAM149A | GFRA2 | HOXB3 | KIF20B |
| ALS2CR8 | AURKA | CAD | CD99L2 | COL6A6 | DNAH14 | FAM160A2 | GGT6 | HTRA2 | KIF27 |
| AMER3 | AVPI1 | CADM1 | CDH19 | COPB2 | DNAJC6 | FAM166B | GHDC | HUWE1 | KIF3C |
| AMICA1 | AXDND1 | CALML4 | CDHR5 | CP | DNASE1L3 | FAM189A2 | GIGYF2 | HVCN1 | KIF7 |
| ANGPTL1 | B3GAT1 | CAMKV | CDK10 | CPEB1 | DPP8 | FAM24B-CUZD1 | GINS3 | ICA1L | KIFC1 |
| ANGPTL6 | BBIP1 | CAMTA2 | CDKN2A | CPEB2 | DPY19L2P2 | FAM46A | GJB6 | IFI44L | KIR2DL4 |

**S3 Table (cont’d) – List of genes for which AS is uniquely dysregulated in EBV-negative GC (Tumor, no virus)**

| TNoV/NNoV | | | | | | | | | |
|---|---|---|---|---|---|---|---|---|---|
| KIR3DL1 | LRP6 | MLH1 | NEDD4 | OGDHL | PIK3CG | PROM2 | REG3A | SCYL1 | SLC38A9 |
| KLF6 | LRRC20 | MLIP | NEK2 | OGFOD2 | PIK3R5 | PRPSAP1 | RFFL | SEC16B | SLC41A3 |
| KLHDC1 | LRRC28 | MLLT10 | NELL2 | OIT3 | PKNOX2 | PRR4 | RHBG | SEC31B | SLC45A2 |
| KLHDC4 | LRRC43 | MLXIPL | NEU4 | OLR1 | PLA2G12B | PRRG1 | RHCG | SELP | SLC4A3 |
| KLHDC8A | LRRK1 | MORC4 | NFASC | OS9 | PLA2G2A | PRSS37 | RIMS2 | SEMA4A | SLC4A7 |
| KLHL32 | LSM5 | MORF4L1 | NHLRC3 | OSBPL8 | PLA2G4F | PSMC1 | RIN1 | SEMA5B | SLC5A11 |
| KLK12 | LTBP3 | MPDZ | NHSL1 | OTUB1 | PLAU | PSTPIP2 | RNASEK-C17orf49 | SEMA6B | SLC5A9 |
| KLK13 | LTK | MPHOSPH9 | NLN | P2RX2 | PLB1 | PTAFR | RNF150 | SEMA6D | SLC6A10P |
| KLK8 | LUZP2 | MPPED2 | NMNAT3 | PADI1 | PLD3 | PTCH1 | RNF40 | SEPP1 | SLC6A6 |
| KMT2E | LY6K | MROH1 | NMT1 | PALM | PLEKHA2 | PTGES3 | RNFT1 | SERPINB13 | SLC8A2 |
| KRAS | LYSMD4 | MRPL42 | NOL6 | PAPLN | PLEKHA6 | PTK6 | RNFT2 | SERPINF2 | SLC8A3 |
| KREMEN1 | M1AP | MRPL43 | NPAS1 | PAPSS2 | PLEKHG3 | PTK7 | RNLS | SEZ6L | SLC9B2 |
| KRIT1 | MAEA | MRPL47 | NPC1 | PARD3 | PLS3 | PTPN14 | RPAIN | SGCA | SLFNL1 |
| KRT15 | MAL | MRPL52 | NPC1L1 | PARP8 | PLSCR3 | PTPN20B | RPE | SH2D1B | SMARCE1 |
| L3MBTL4 | MAP2 | MRPS11 | NPHP3 | PAX5 | PMEPA1 | PTPRA | RPL12 | SH3BP4 | SMYD1 |
| LAX1 | MAP3K9 | MRPS12 | NPL | PAX8 | PMS1 | PTPRK | RPL17 | SH3GL3 | SNAP91 |
| LDB3 | MAP4K3 | MRPS25 | NPM1 | PBXIP1 | PNLIPRP1 | PTPRVP | RPL9 | SH3GLB1 | SNAPC3 |
| LEF1 | MAP7D2 | MS4A14 | NPM2 | PCA3 | PNLIPRP2 | PUM1 | RPS9 | SIGMAR1 | SNRPN |
| LGALS9B | MAPK10 | MSS51 | NPNT | PCBP3 | PNPLA7 | PUS7 | RREB1 | SLC11A1 | SNURF-SNRPN |
| LGMN | MAPK7 | MTFP1 | NPPA-AS1 | PCDH17 | PODNL1 | PUS7L | RRN3P3 | SLC13A2 | SNX11 |
| LGR5 | MAPKAPK5-AS1 | MTG2 | NR1H4 | PCDH18 | POFUT1 | PVRL4 | RSPO2 | SLC13A4 | SNX17 |
| LIG1 | MAPKBP1 | MTHFD2 | NR1I3 | PCK2 | POGZ | PWWP2A | RSPRY1 | SLC16A6 | SNX5 |
| LILRB2 | MATN3 | MTHFSD | NR2C1 | PCSK4 | POLM | PXK | RUNDC3B | SLC17A3 | SOSTDC1 |
| LILRB5 | MATR3 | MTUS1 | NR3C1 | PCYOX1L | POMGNT1 | RAB11FIP1 | RUNX1 | SLC17A7 | SOX6 |
| LIN7A | MCCC1 | MVP | NR3C2 | PDE2A | POMT1 | RAB7L1 | RUNX2 | SLC17A8 | SP3 |
| LINC00470 | MCM10 | MYB | NRAP | PDE8B | POSTN | RAC1 | RUSC1 | SLC22A7 | SPAG11B |
| LINC00894 | MCM4 | MYBL1 | NRD1 | PDE9A | PPFIBP2 | RAD51AP1 | SALL4 | SLC23A3 | SPATA6L |
| LINC00907 | MCMDC2 | MYBPHL | NRG3 | PDK1 | PPIP5K2 | RADIL | SAMD9L | SLC25A13 | SPINK5 |
| LMAN2L | MCTP2 | MYD88 | NRG4 | PDLIM3 | PPP1CB | RANBP9 | SAR1A | SLC25A14 | SPIRE2 |
| LMO3 | MDH1B | MYH14 | NRXN1 | PDSS2 | PPP1R1B | RAP1GAP | SCAF8 | SLC25A45 | SPNS1 |
| LOC100131060 | MED24 | MYO5C | NSG1 | PDXDC1 | PPP1R9A | RASA1 | SCAND2P | SLC26A10 | SPP1 |
| LOC100287534 | MEGF8 | MYOM1 | NSMF | PDZD4 | PPP2R5D | RASAL2-AS1 | SCARA5 | SLC26A6 | SREBF1 |
| LOC100506990 | MELK | MYOT | NUCB2 | PEX11A | PPP2R5E | RASSF8 | SCARB1 | SLC28A1 | SRGAP2 |
| LOC399815 | MEMO1 | NAALADL1 | NUDT13 | PFKFB3 | PPRC1 | RBBP5 | SCEL | SLC2A2 | STAG3L1 |
| LOC400927 | METTL16 | NAPB | NUMA1 | PGAP2 | PPT1 | RBM26 | SCFD1 | SLC2A5 | STARD7 |
| LOC643201 | MFSD12 | NBEA | NUMB | PGPEP1 | PPT2-EGFL8 | RBM47 | SCG3 | SLC2A6 | STAU1 |
| LOC728323 | MFSD4 | NBEAP1 | NUP50 | PGR | PPWD1 | RBM6 | SCMH1 | SLC30A10 | STK32C |
| LONRF1 | MGC4836 | NCAM1 | NUP62CL | PHYHD1 | PRDM5 | RC3H1 | SCN7A | SLC35E2B | STT3A |
| LOXL3 | MGEA5 | NDRG1 | NXF2 | PHYKPL | PRKD2 | RCBTB2 | SCNN1D | SLC35F2 | STX5 |
| LPAR6 | MICAL2 | NEDD1 | OBSCN | PIGG | PRNP | RCCD1 | SCTR | SLC37A2 | STXBP5 |

## S3 Table (cont'd) – List of genes for which AS is uniquely dysregulated in EBV-negative GC (Tumor, no virus)

| TNoV/NNoV | | | | | | | | | |
|---|---|---|---|---|---|---|---|---|---|
| STXBP6 | TBCE | TIE1 | TMEM260 | TRABD2A | TTYH3 | WASF3 | YTHDF3 | ZKSCAN7 | ZNF544 |
| SULT1A1 | TBXAS1 | TINF2 | TMEM39B | TRAF3IP3 | TUBD1 | WBP2 | ZBED3-AS1 | ZMIZ1 | ZNF566 |
| SUN2 | TCEA1 | TLE1 | TMPRSS13 | TRAF4 | TVP23A | WDR17 | ZBED5 | ZMYM6 | ZNF571-AS1 |
| SUPT7L | TCEA3 | TLR2 | TMPRSS5 | TREH | TVP23C-CDRT4 | WDR47 | ZBTB44 | ZNF12 | ZNF584 |
| SVEP1 | TCF12 | TMBIM1 | TNC | TRIB2 | TXLNG2P | WDR49 | ZBTB8A | ZNF131 | ZNF596 |
| SYBU | TCF7 | TMEM106A | TNFRSF17 | TRIM26 | U2AF1 | WDR6 | ZCCHC17 | ZNF135 | ZNF695 |
| SYCP2L | TCP11L1 | TMEM107 | TNFRSF25 | TRIM33 | UBXN2A | WDR65 | ZCCHC6 | ZNF146 | ZNF718 |
| SYN1 | TDRD1 | TMEM126B | TNFRSF8 | TRIM46 | UGT3A2 | WDR67 | ZCCHC7 | ZNF160 | ZNF780B |
| SYN2 | TDRKH | TMEM129 | TNIP3 | TRIM47 | URGCP | WHAMMP2 | ZCWPW2 | ZNF181 | ZNF781 |
| SYNE2 | TEAD2 | TMEM130 | TNNT2 | TRIM6 | USHBP1 | WISP2 | ZEB1 | ZNF211 | ZNF815P |
| SYPL2 | TENM2 | TMEM136 | TOM1L2 | TRIO | USP47 | XAF1 | ZEB2 | ZNF226 | ZNF85 |
| TAC3 | TESPA1 | TMEM150B | TOMM40L | TRO | VARS2 | XAGE1B | ZFAND1 | ZNF250 | ZSCAN2 |
| TAF1C | TFCP2L1 | TMEM169 | TOPORS | TRPC4 | VIT | XAGE1C | ZFAND5 | ZNF268 | ZSCAN23 |
| TAGAP | TFEB | TMEM175 | TP53 | TRPS1 | VLDLR | XAGE1D | ZFAT | ZNF274 | ZSCAN26 |
| TAOK2 | TGFB2 | TMEM180 | TP63 | TRPV4 | VMO1 | YAP1 | ZFP37 | ZNF3 | ZSCAN32 |
| TBC1D30 | THAP6 | TMEM189 | TPM3 | TTC8 | VMP1 | YJEFN3 | ZFYVE28 | ZNF382 | ZZZ3 |
| TBC1D3G | THBS3 | TMEM241 | TPO | TTI2 | VPS53 | YME1L1 | ZHX3 | ZNF410 | |
| TBC1D3H | THSD1 | TMEM255A | TPT1 | TTLL6 | VRK2 | YPEL5 | ZKSCAN5 | ZNF541 | |
