# Supplementary material for: Transcriptome-wide analysis of alternative RNA splicing events in Epstein-Barr virus-associated gastric carcinomas
Source: PLoS One. 2017 May 11;12(5):e0176880. doi: 10.1371/journal.pone.0176880 (PMC5426614; doi:10.1371/journal.pone.0176880)
Supplement: S4 Table — (PDF) [file pone.0176880.s004.pdf]

S4 Table – List of splicing factors that co-purify with EBNA1

Gene	Intensity	Ratio
hnRNP M	611450000	70
hnRNP H1	425050000	51
hnRNP U	302890000	32
hnRNP K	133310000	31
hnRNP C	237640000	29
DHX15	133450000	18
SFPQ	43327000	13
PRPF8	12030000	7
SF3B1	4349800	2
DHX30	10107000	2
