# Supplementary material for: Transcriptome-wide analysis of alternative RNA splicing events in Epstein-Barr virus-associated gastric carcinomas
Source: PLoS One. 2017 May 11;12(5):e0176880. doi: 10.1371/journal.pone.0176880 (PMC5426614; doi:10.1371/journal.pone.0176880)
Supplement: S1 Fig — Distribution of Delta PSI values in GC. (PDF) [file pone.0176880.s005.pdf]

|ΔPSI|	TNoV/NNoV	TEBV/NNoV
[10-20[	1907	768
[20-30[	600	331
[30-40[	229	103
[40-50[	73	56
[50-60[	36	26
[60-70[	8	6
[70-80[	5	4
[80-90[	5	1
[90-100]	0	2
