# Supplementary material for: Transcriptome-wide analysis of alternative RNA splicing events in Epstein-Barr virus-associated gastric carcinomas
Source: PLoS One. 2017 May 11;12(5):e0176880. doi: 10.1371/journal.pone.0176880 (PMC5426614; doi:10.1371/journal.pone.0176880)
Supplement: S2 Fig — Overrepresentation of biological processes was analyzed for the differential ASEs associated with EBV-negative GC, EBVaGC, and cells expressing the EBNA1 protein. The first column highlights the name of the biological process, the second column displays the total corresponding number of genes in the database (PANTHER), the third column highlights the number of genes from the searched sample, the fourth column displays the expected number of genes in a normal cell, and the fifth column displays the fold-enrichment. The p-value associated with each overrepresentation is also displayed (last column). (PDF) [file pone.0176880.s006.pdf]

EBV-negative GC

PANTHER GO-Slim Biological Process	#	#	expected	Fold Enrichment	P value
heart development	181	31	14.41	2.15	2.02E-02
vesicle-mediated transport	895	121	71.25	1.70	5.13E-06
↳transport	2473	268	196.88	1.36	3.20E-05
↳localization	2607	290	207.54	1.40	7.44E-07
cellular component movement	476	64	37.89	1.69	1.23E-02
↳cellular process	6708	627	534.02	1.17	1.77E-04
immune system process	1391	157	110.74	1.42	2.17E-03
intracellular protein transport	1052	118	83.75	1.41	3.60E-02
↳protein transport	1082	125	86.14	1.45	6.77E-03
primary metabolic process	6825	623	543.34	1.15	4.59E-03
↳metabolic process	8247	762	656.54	1.16	1.84E-05

EBVaGC

PANTHER GO-Slim Biological Process	#	#	expected	Fold Enrichment	P value
cellular component movement	476	42	19.69	2.13	1.37E-03
vesicle-mediated transport	895	64	37.02	1.73	5.07E-03
cellular component organization	1206	80	49.89	1.60	6.58E-03
↳cellular component organization or biogenesis	1316	88	54.44	1.62	1.92E-03
metabolic process	8247	394	341.15	1.15	3.22E-02
Unclassified	8629	304	356.95	.85	0.00E00

EBNA1-expressing cells

PANTHER GO-Slim Biological Process	#	#	expected	Fold Enrichment	P value
response to stimulus	2170	25	11.68	2.14	4.21E-02
