# Supplementary figures and images for: Transcriptome-wide analysis of alternative RNA splicing events in Epstein-Barr virus-associated gastric carcinomas
Source: PLoS One. 2017 May 11;12(5):e0176880. doi: 10.1371/journal.pone.0176880 (PMC5426614; doi:10.1371/journal.pone.0176880)

## Transcription factors

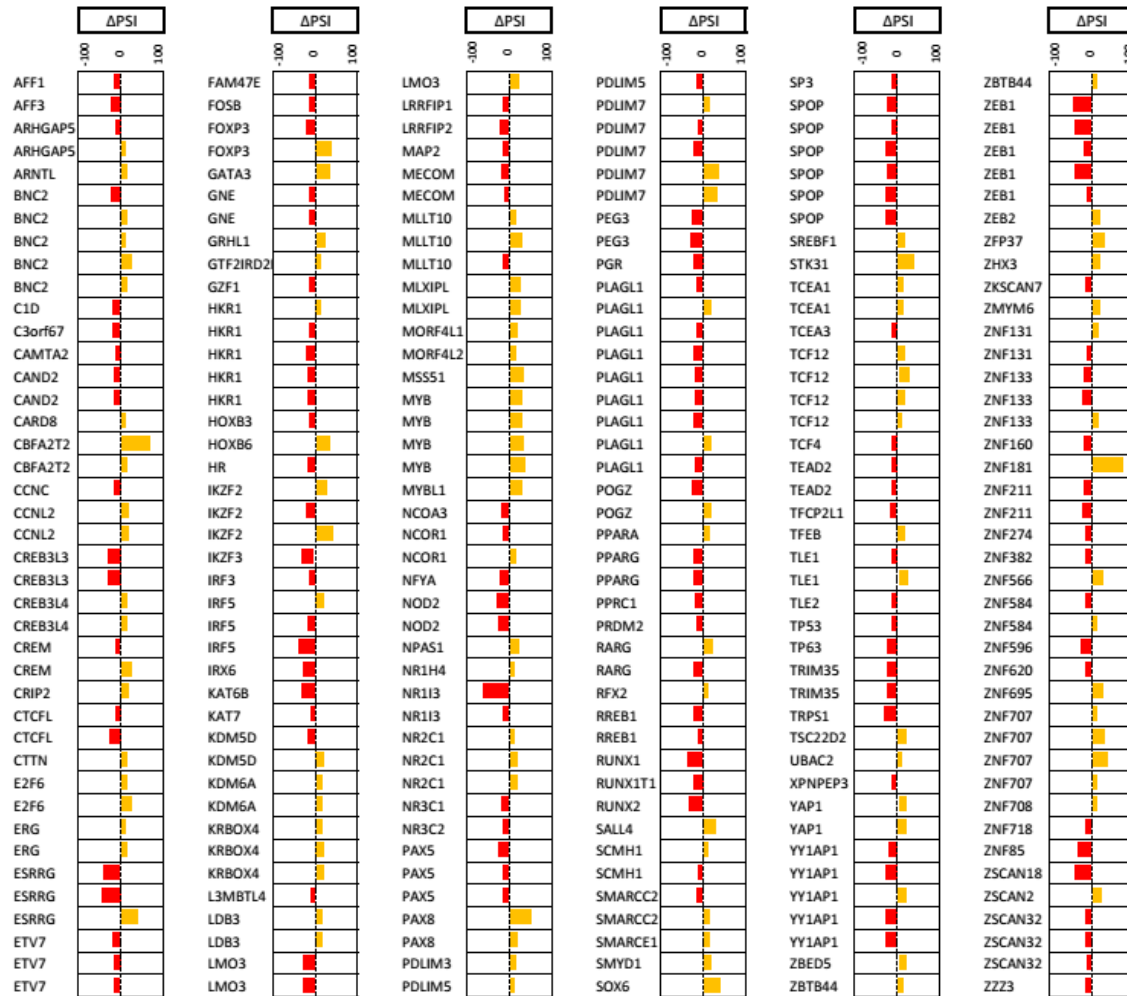

Supplement: S3 Fig — The cDNAs acquired from gastric tissues were analyzed by PCR using specific primers to detect both isoforms of the transcripts encoded by the CARN14, PLA2G4F, S100A1, SLC52A1, and SOGA2 genes. Capillary electrophoregrams of the PCR reactions are presented. The positions and the amplitude of the detected amplicons are indicated. The positions of the internal markers are also shown. (PDF) [file pone.0176880.s007.pdf]

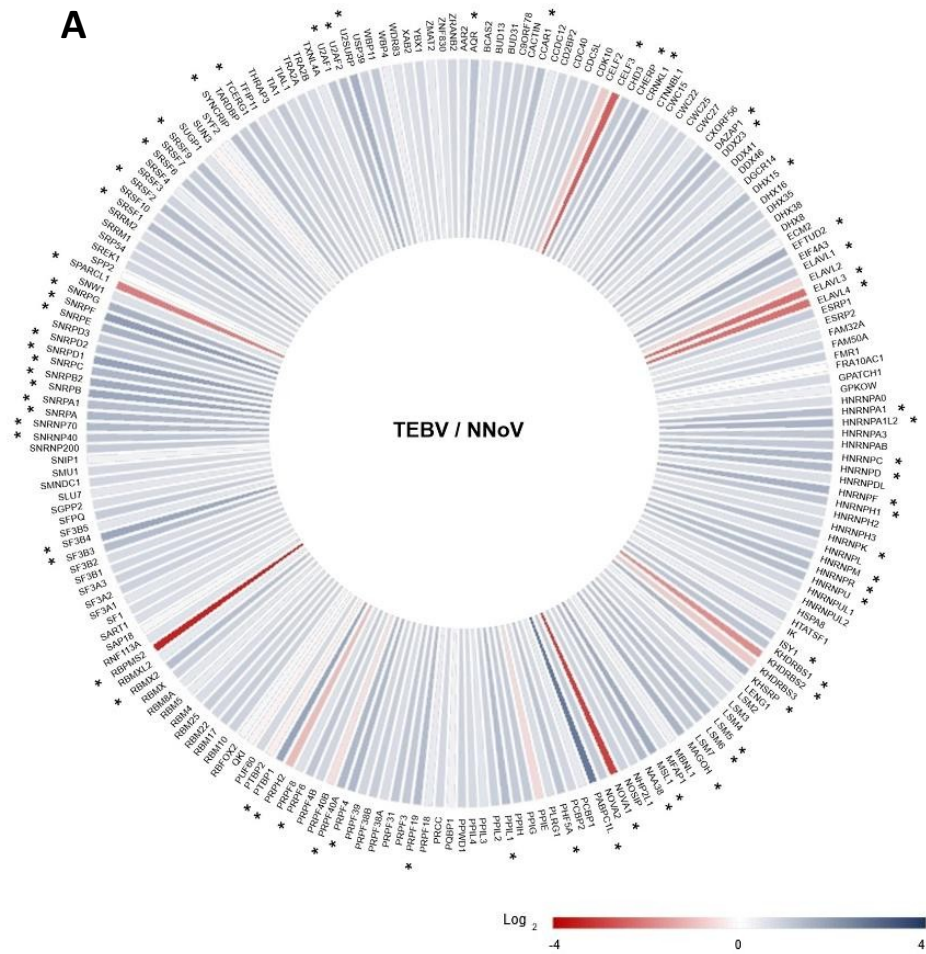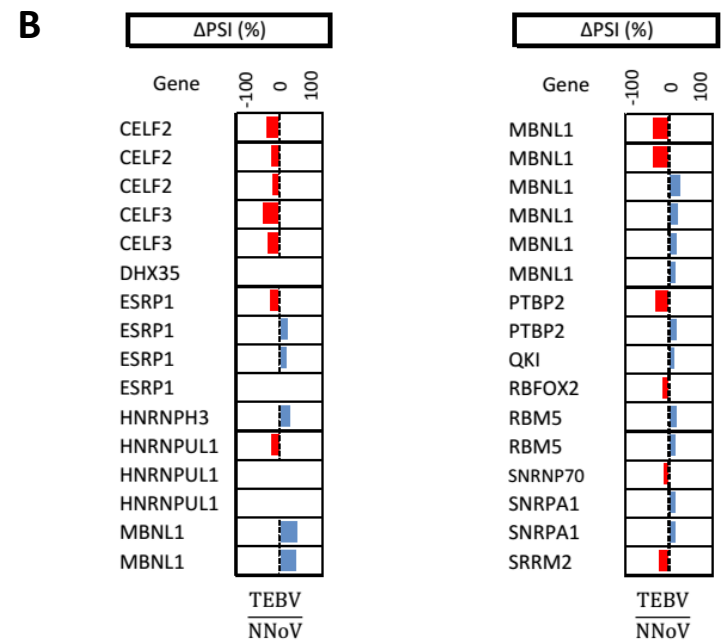

Supplement: S4 Fig — (A) Iris Graph representing the expression profile of splicing factors for EBVaGC. Differences in gene expression levels are shown on a logarithmic color scale (Log2), from red (negative changes in expression) to blue (increase in gene expression). The expression of proteins involved in splicing modulated by more than 2-fold is indicated by an asterisk. (B) Misregulation of splicing factors alternative splicing in GC (TEBV, Tumors with EBV). The delta PSI values are represented in red (negative delta PSI values) and in blue (positive delta PSI values).) (PDF) [file pone.0176880.s008.pdf]
