# Supplementary material for: Transcriptome-wide analysis of alternative RNA splicing events in Epstein-Barr virus-associated gastric carcinomas
Source: PLoS One. 2017 May 11;12(5):e0176880. doi: 10.1371/journal.pone.0176880 (PMC5426614; doi:10.1371/journal.pone.0176880)
Supplement: S5 Fig — Differences in gene expression levels of proteins involved in splicing are shown. The figure only shows the splicing factors for which the expression level varied by more than 2-fold. A logarithmic color scale (Log2) is used. Red indicates negative changes in expression and blue indicates increase in gene expression. (PDF) [file pone.0176880.s009.pdf]

TNoV/NNoV		
Gene	Log2	q-value
CCAR1	1.00	3.6668E-28
DHX15	1.07	2.6879E-30
EFTUD2	1.24	5.3925E-31
ELAVL3	-1.73	0.00691799
ELAVL4	-1.06	0.03160062
HNRNPA1L2	1.07	7.0366E-37
HNRNPF	1.08	1.3269E-33
KHDRBS3	1.17	1.8774E-07
LSM5	1.01	5.5924E-22
NAA38	1.07	9.182E-14
NOVA1	-1.35	0.00341839
PABPC1L	2.96	2.3362E-28
PPIL1	1.19	9.4054E-35
PRPF3	1.01	1.1643E-17
PRPF4	1.17	8.4559E-37
PRPF40A	1.07	2.547E-24
PTBP1	1.42	9.3948E-25
RBPMS2	-2.08	0.00093872
SF3B3	1.06	5.3668E-16
SF3B4	1.29	1.1709E-15
SNRPA1	1.11	9.8491E-44
SNRPB	1.17	9.8242E-33
SNRPB2	1.23	1.0417E-30
SNRPD1	1.59	5.6632E-40
SNRPE	1.11	1.5001E-23
SNRPF	1.18	6.4859E-38
SNRPG	1.28	1.4453E-28
SPARCL1	-1.06	0.00662097
SRSF1	1.02	1.3896E-29
SRSF10	1.05	2.3152E-15
SRSF6	1.01	1.8993E-22
SUN3	3.18	0.01407861
SYNCRIP	1.05	1.5498E-21
U2SURP	1.23	3.6222E-21

TEBV/NNoV					
Gene	Log2	q-value	Gene	Log2	q-value
AQR	1.04	5.969E-06	PPIL1	1.18	2.0567E-09
CCAR1	1.09	3.6233E-07	PRPF19	1.21	1.9088E-08
CELF3	-2.51	6.4645E-06	PRPF4	1.24	2.1604E-08
CHERP	1.06	2.4545E-08	PRPF40A	1.25	3.3274E-07
CRNKL1	1.18	3.7171E-07	PRPF6	1.00	2.863E-07
DAZAP1	1.14	9.0004E-09	PRPH2	-1.07	0.00365944
DDX23	1.06	2.4326E-07	PTBP1	1.61	1.9812E-06
DHX15	1.13	1.4054E-07	RBMX2	1.12	2.3309E-08
EFTUD2	1.41	1.4112E-06	RBPMS2	-3.48	0.00031047
ELAVL1	1.02	5.3535E-11	SF3B3	1.15	2.001E-07
ELAVL3	-2.26	0.00537376	SF3B4	1.57	5.6026E-05
ELAVL4	-2.21	0.00497492	SNRNP40	1.02	6.8481E-10
HNRNPA1	1.19	1.7001E-07	SNRNP70	1.24	9.1745E-10
HNRNPA1L2	1.38	4.5011E-08	SNRPA	1.27	1.9868E-09
HNRNPC	1.28	8.1328E-06	SNRPA1	1.16	5.2256E-07
HNRNPD	1.23	1.2504E-10	SNRPB	1.56	1.1481E-09
HNRNPF	1.38	8.0934E-09	SNRPB2	1.50	1.2002E-07
HNRNPH1	1.02	3.5424E-08	SNRPC	1.27	9.8292E-11
HNRNPK	1.12	4.7844E-08	SNRPD1	1.65	9.7589E-09
HNRNPM	1.07	5.198E-09	SNRPD2	1.14	1.7509E-08
HNRNPR	1.18	1.9782E-08	SNRPE	1.38	3.4792E-08
HNRNPU	1.14	2.1479E-09	SNRPF	1.77	9.0897E-09
ISY1	1.04	2.5824E-07	SNRPG	1.36	1.6073E-07
KHDRBS1	1.04	6.8239E-10	SPARCL1	-2.02	0.00080958
KHDRBS2	-1.78	0.0112962	SRSF1	1.19	1.1139E-07
KHSRP	1.29	4.3961E-10	SRSF2	1.15	1.7509E-08
LSM5	1.17	1.8123E-08	SRSF6	1.03	2.6535E-07
LSM6	1.06	8.4922E-09	SRSF9	1.12	1.1252E-12
MAGOH	1.32	9.7091E-09	SYNCRIP	1.14	9.7378E-07
MSL1	1.48	0.01486311	TCERG1	1.03	1.1237E-07
NAA38	1.19	2.8367E-09	U2AF1	1.01	2.8907E-08
NOVA1	-3.00	0.00025888	U2AF2	1.16	9.1705E-09
PABPC1L	2.40	3.766E-07	U2SURP	1.40	3.766E-07
PHF5A	1.04	1.2017E-07			
